# NK cells produce high levels of IL‐10 early after allogeneic stem cell transplantation and suppress development of acute GVHD

**DOI:** 10.1002/eji.201747134

**Published:** 2017-10-27

**Authors:** Yuen Ling Tracey Chan, Jianmin Zuo, Charlotte Inman, Wayne Croft, Jusnara Begum, Joanne Croudace, Francesca Kinsella, Luke Maggs, Sandeep Nagra, Jane Nunnick, Ben Abbotts, Charles Craddock, Ram Malladi, Paul Moss

**Affiliations:** ^1^ Institute of Immunology and Immunotherapy University of Birmingham UK; ^2^ Centre for Computational Biology University of Birmingham UK; ^3^ Birmingham Health Partners Centre for Clinical Haematology Queen Elizabeth Hospital Birmingham UK

**Keywords:** Allo‐SCT, GVHD, IL‐10, NK cells, T cells depleted

## Abstract

Natural killer (NK) cells rapidly reconstitute following allogeneic stem cell transplantation (allo‐SCT), at the time when alloreactive T cell immunity is being established. We investigated very early NK cell reconstitution in 82 patients following T cell‐depleted allo‐SCT. NK cell number rapidly increased, exceeding T cell reconstitution such that the NK:T cell ratio was over 40 by day 14. NK cells at day 14 (NK‐14) were donor‐derived, intensely proliferating and expressed chemokine receptors targeted to lymphoid and peripheral tissue. Spontaneous production of the immunoregulatory cytokine IL‐10 was observed in over 70% of cells and transcription of cytokines and growth factors was augmented. NK‐14 cell number was inversely correlated with the incidence of grade II‐IV acute graft versus host disease (GVHD). These findings reveal that robust reconstitution of immunoregulatory NK cells by day 14 after allo‐SCT is an important determinant of the clinical outcome, suggesting that NK cells may suppress the development of the T cell‐mediated alloreactive immune response through production of IL‐10.

## Introduction

Over 30,000 patients undergo allogeneic stem cell transplantation (allo‐SCT) each year, the great majority as a treatment for haemopoietic malignancy 1. The curative potential of allo‐SCT is mediated by the ‘graft versus leukemia’ effect (GVL), predominantly mediated by the alloreactive donor T cell response. However, this immune response can also lead to severe tissue damage in the patient through the action of acute GVHD 2, 3.

NK cells are an important component of the innate cellular immune response and exhibit strong cytolytic and cytokine activity against tumour cells and virally‐infected cells 4, 5. However, NK cells also have important regulatory functions 6 and localise with haemopoietic cells within secondary lymphoid compartments and at sites of inflammation 7, 8, where they can modulate the adaptive T cell response through cytokine release, cytotoxicity or promotion of antigen cross‐presentation to T cells 8.

NK cells reconstitute early following allo‐SCT 9, 10 and high NK cell number at day 30–60 after transplantation is associated with a reduced rate of disease relapse and improved overall survival 11, 12, 13. Furthermore, infusion of NK cells protects against acute GVHD whilst retaining GVL in murine models 14, 15. These observations suggest that NK cells have the potential to suppress the development of T cell mediated acute GVHD through undefined mechanisms without compromising the beneficial effect of allo‐SCT. Indeed, robust NK cell reconstitution has been seen to predict for a lower risk of acute GVHD 12, 13, 14.

We undertook a detailed assessment of the function and phenotype of NK cells within the first 2 weeks after transplant, and correlated this with the clinical outcome. We demonstrate that donor NK cells proliferate intensively during this period and become targeted to secondary lymphoid and peripheral tissues through expression of a range of chemokine receptors. Moreover they exhibit very high expression of IL‐10 in a pattern that mirrors the ‘proliferation‐induced conditioning’ observed within murine NK cells and which acts to suppress adaptive immunity. Importantly, higher numbers of NK cells at 14 days after transplant are associated with a reduced risk of acute GVHD.

## Results

### NK cells reconstitute rapidly following allo‐SCT

The pattern of NK cell reconstitution was examined in a cohort of 82 consecutive patients that underwent allo‐SCT using mobilised peripheral blood (Table 1). 87% of patients received a RIC transplant whereas 13% underwent myeloablative conditioning. 32% of patients received HLA‐identical transplants from siblings and the remaining 68% received transplants from unrelated donors. All patients received T cell depletion, 13% with ATG and 87% with alemtuzumab, and both ciclosporin and methotrexate were used for GVHD prophylaxis according to standard protocols. At 100 days the cumulative incidence of grade II‐IV acute GVHD was 25% and the incidence of chronic GVHD, relapse, transplant‐related mortality (TRM) and overall survival at 3 years was 29, 30, 20 and 53% respectively (Table 2).

**Table 1:** Patient and transplant characteristics and GVHD prophylaxis

		Number	%
**Patient details**			
Age at transplant	Median years (range) (IQR)	52.5 (17–71)	
		(44‐61)	
Sex	Male	52	63.4
	Female	30	36.6
Diagnosis	AML/MDS	43	52.4
	NHL	13	15.9
	ALL	12	14.6
	AA	4	4.9
	MF	3	3.7
	HL	3	3.7
	CLL	2	2.4
	MPD	2	2.4
Sorror score	0	63	76.8
	≥ 1	19	23.2
Disease risk	Low	14	17.1
Index	Intermediate	36	43.9
	High	29	35.4
	Very High	3	3.7
**Transplant details**			
Intensity	Reduced intensity	71	86.6
	Myeloablative	11	13.4
CMV at risk	Yes	67	81.7
Male female mismatch	Yes	20	24.4
Donor	Unrelated	56	68.3
	Sibling	26	31.7
HLA mismatch	None	68	82.9
	One	14	17.1
Stem cell source	PBSC	82	100
**GVHD prophylaxis**			
T cell depletion	ATG	11	13.4
	Alemtuzumab	71	86.6
Methotrexate	Yes	14	17.1
Ciclosporin	Yes	82	100

AML, acute myeloid leukaemia; MDS, myelodysplastic syndrome; NHL, non‐Hodgkin's lymphoma; ALL, acute lymphoblastic leukaemia; AA, aplastic anaemia; MF, myelofibrosis; HL, Hodgkin's lymphoma; CLL, chronic lymphocytic leukaemia; MPD, myeloproliferative disease; ATG, anti‐thymocyte globulin; MF mismatch, male female mismatch; HLA, human leukocyte antigen; PBSC, peripheral blood stem cell. CMV at risk is defined as either patient or donor being CMV positive.

**Table 2:** Patient outcomes

	Cumulative incidence or overall survival (95% CI)
Grade II‐IV acute GVHD at 100 days	25% (16–35%)
Chronic GVHD at 3 years	29% (20–40%)
Relapse at 3 years	30% (20–40%)
TRM at 3 years	20% (11–29%)
Survival at 3 years	53% (39–73%)

**Figure 1:**
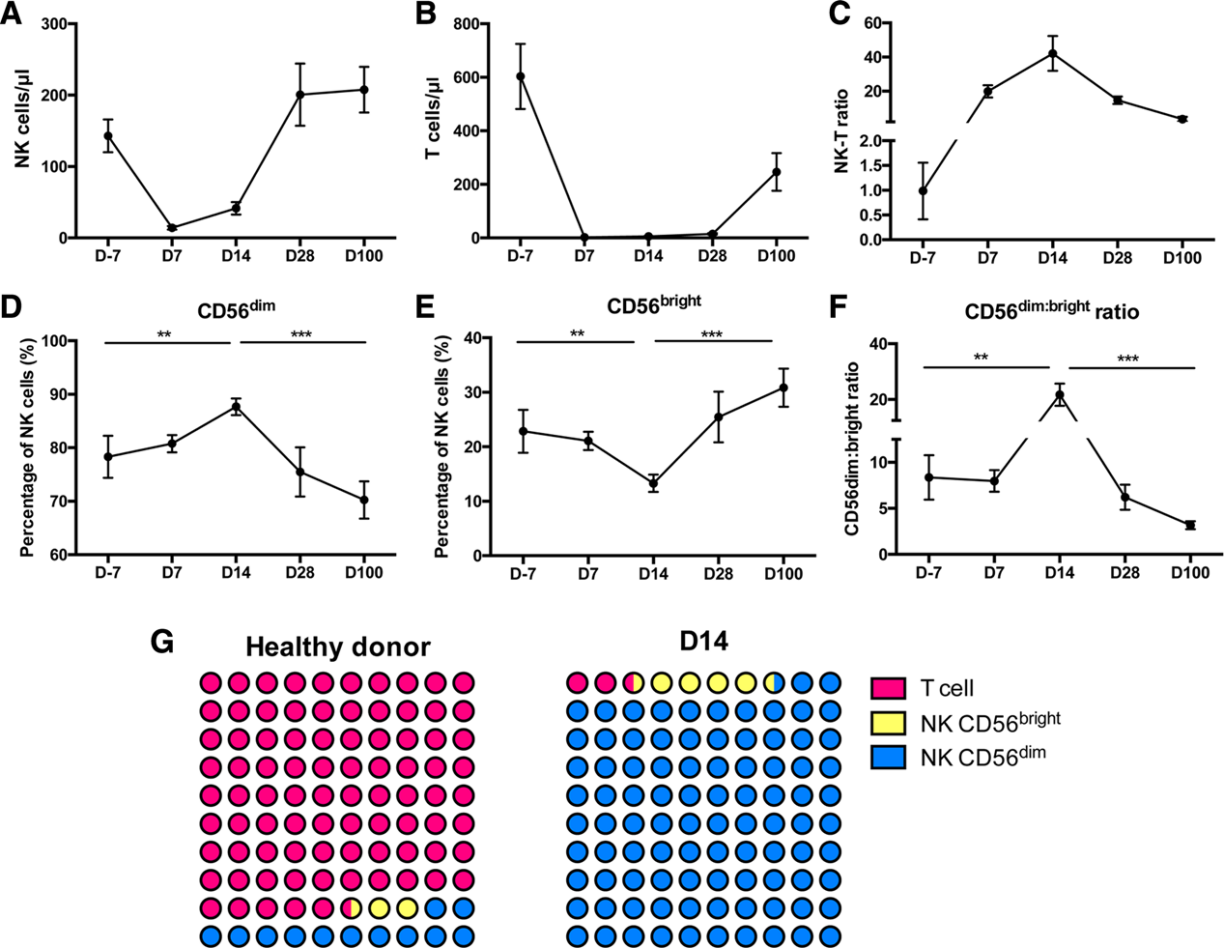
The reconstitution of NK cells in the very early period following allo‐SCT. PBMCs were isolated by density centrifugation. Mononuclear cells were incubated with surface antibodies on ice for 20 min with Propidium iodide (PI) as viability dye. The percentage of the different cell populations was assessed by Flow cytometry. (A) Reconstitution of the number of NK cells in the first 100 days following allo‐SCT (B) Reconstitution of the number of T cells in the first 100 days following allo‐SCT. (C) Ratio of NK cell: T cell number up to 100 days. (D) CD56^dim^ NK cells and (E) CD56^bright^ NK cells expressed as a percentage of the total NK cell population in the first 100 days following allo‐SCT. (F) The ratio of CD56^dim^ NK cells: CD56^bright^ NK cells plotted following allo‐SCT. Data are pooled from 20 to 82 independent patients staining . All graphs depict mean and error bars represent the standard error of the mean. Day ‐7 (*n* = 20), Day 7 (*n* = 82), Day 14 (*n* = 82), Day 28 (*n* = 20), Day 100 (*n* = 23). ^**^ *p* < 0.01, ^***^ *p* < 0.005, two‐tailed, unpaired Mann–Whitney test. (G) Diagrammatic representation contrasting the ratio of CD56^bright^ and CD56^dim^ NK cells compared to T cells at day 14 following allo‐SCT in comparison to healthy donors.

Flow cytometry was used to define the reconstitution of NK and T cells up to 100 days following allo‐SCT (Gating strategy in Fig. S1). The number of NK and T cells fell markedly in the first week following transplantation (Fig. 1A and B) but subsequent recovery was dramatic in NK cells, which reached pre‐transplant (D‐7) levels within 28 days (Fig. 1A). In contrast, T lymphocytes reconstituted to only 40% of pre‐transplant levels by day 100 (Fig. 1B). The ratio of NK to T cells (NK:T) was approximately 1 prior to transplantation, higher than the NK:T ratio of 0.17 in healthy individuals 16. The NK:T ratio further increased dramatically after transplant such that NK cells outnumbered T cells by over 40 fold at day 14. The NK:T ratio remained higher than a healthy individual even at 100 days post‐transplant (Fig. 1C).

CD56 expression is used to distinguish two subpopulations of NK cells with functional differences 4 and CD56^bright^ NK cells are regarded as less cytotoxic and less mature in comparison to CD56^dim^ populations. CD56^dim^ NK cells comprised 78% of the total NK cell population prior to therapy, increasing slightly to 88% by day 14 before dropping to 75% at day 28 (Fig. 1D). In contrast, CD56^bright^ NK cells (Fig. 1E) comprised 22% of the NK repertoire before transplantation, falls to 12% at day 14 and then expands to 25% at day 28 and 31% by day 100. As such the CD56^dim^:CD56^bright^ ratio (Fig. 1F) of 8.4 prior to treatment rose to 22 by D14 reflecting an increase in the proportion of mature NK cells. The dramatic difference in the proportion of CD56^bright^ NK cells and CD56^dim^ NK cells relative to T cells at day 14 compared to healthy donors is displayed in Fig. 1G. Interestingly, in an analysis T cell replete transplants the CD56^bright^ NK cell subset at day 14 was significantly larger than that seen in TCD transplants at D14 (*p* < 0.001) (Supporting Information Fig. 2).

### NK cells at day 14 express higher levels of NKG2A and reduced expression of NKG2C

**Figure 2:**
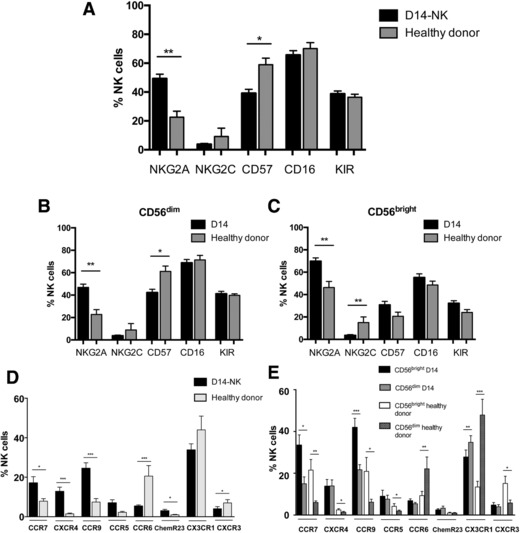
Phenotypic changes seen on NK cells during very early reconstitution after allo‐SCT. PBMCs were isolated by density centrifugation. Mononuclear cells were incubated with surface antibodies on ice for 20 min with Propidium iodide (PI) as viability dye. The percentage of the different cell populations was assessed by flow cytometry. (A) Cell surface marker expression on NK cells at day 14 after allo‐SCT (NK‐14) and NK cells within healthy donors. Cell surface marker expression on both the (B) CD56^dim^ and (C) CD56^bright^ NK cell subsets. (D) Chemokine receptor expression on the total NK cell population as well as (E) the CD56^dim^ and CD56^bright^ NK cell subsets. (A–C): NK‐14 (*n* = 82) and healthy donors (*n* = 12). (D and E): NK‐14 (*n* = 32) and healthy donors (*n* = 12). Data are pooled from 12 to 82 independent patients staining. For all graphs, the mean and standard error of the mean are depicted. ^*^ *p* < 0.05, ^**^ *p* < 0.01; ^***^ *p* < 0.005, two‐tailed, Mann–Whitney test for unpaired analyses, Wilcoxon matched‐pairs signed rank test for paired analyses between CD56^bright^ and CD56^dim^ NK cell subsets.

We next went on to examine the phenotype of NK cells at day 14 (NK‐14) relative to the profile of NK cells from healthy donors (Fig. 2A). NKG2A expression was markedly increased on both the CD56^dim^ and CD56^bright^ populations at day 14. In particular, NKG2A expression was observed on 23 and 46% of CD56^dim^ and CD56^bright^ NK cells within healthy donors whereas this increased to 47 and 70% on NK‐14 cells. An inverse relationship was found for the activating receptor NKG2C with higher expression observed for both CD56^dim^ and CD56^bright^ populations in the control group (9 and 15% respectively) compared to our patient cohort (4 and 4%). This increase in expression of NKG2A, and associated reduction in expression of NKG2C, would suggest a more immature phenotype of NK‐14 cells although this is unexpected given the relative expansion of CD56^dim^ cells. Expression of CD57, associated with a terminally differentiated phenotype, showed a differential pattern of staining on NK‐14 cells. The expression of CD57 on CD56^bright^ cells increased from 21% within healthy donors to 31% in the patient group. In contrast, expression of CD57 on CD56^dim^ cells at day 14 decrease from 61% within healthy donors to 43% in the patient group. The expression of CD16 and KIR was similar in patient and healthy donor groups and between CD56^dim^ and CD56^bright^ populations. (Fig. 2B and C).

### Expression of chemokine receptors on NK‐14 cells

We then examined the expression of a range of chemokine receptors that determine the ability of cells to traffic to tissue sites. Analysis included three receptors for homeostatic chemokines (CCR7, CXCR4 and CCR9), receptors for chemokines that guide cells to the gastrointestinal tract (CCR9, CCR5 and CCR6) or skin (ChemR23, CX3CR1 and CXCR3) (Fig. 2D and E). Interestingly, homeostatic chemokine receptor expression was relatively low on NK cells from healthy donors, although CCR7 and CCR9 were expressed on around 20% of CD56^bright^ cells. Expression of receptors for the homeostatic chemokines was markedly increased on NK‐14 cells with the most dramatic increase in the expression of CXCR4, rising from 1% on healthy donor NK cells to 13% of D14‐NK cells. Expression of these three receptors was higher on the CD56^bright^ NK cell subset within healthy donors, and although this differential pattern was largely retained on NK‐14 cells. The overall increase in receptor expression on NK‐14 cells is so great that levels observed on patient CD56^dim^ subsets exceeded values on CD56^bright^ cells from healthy donors in many cases.

More variation was observed in the pattern of expression of receptors for chemokines that guide trafficking to the skin and gastrointestinal tract. In addition to the increase in CCR9, expression of CCR5 was increased from 2% of cells in healthy donors to 7% on NK‐14 samples. In contrast, expression of CCR6, the ligand for MIP‐3α, fell from 21% on healthy donor cells to 5% on NK‐14 populations.

### NK cells at day 14 are derived from the donor and are undergoing intense proliferation

**Figure 3:**
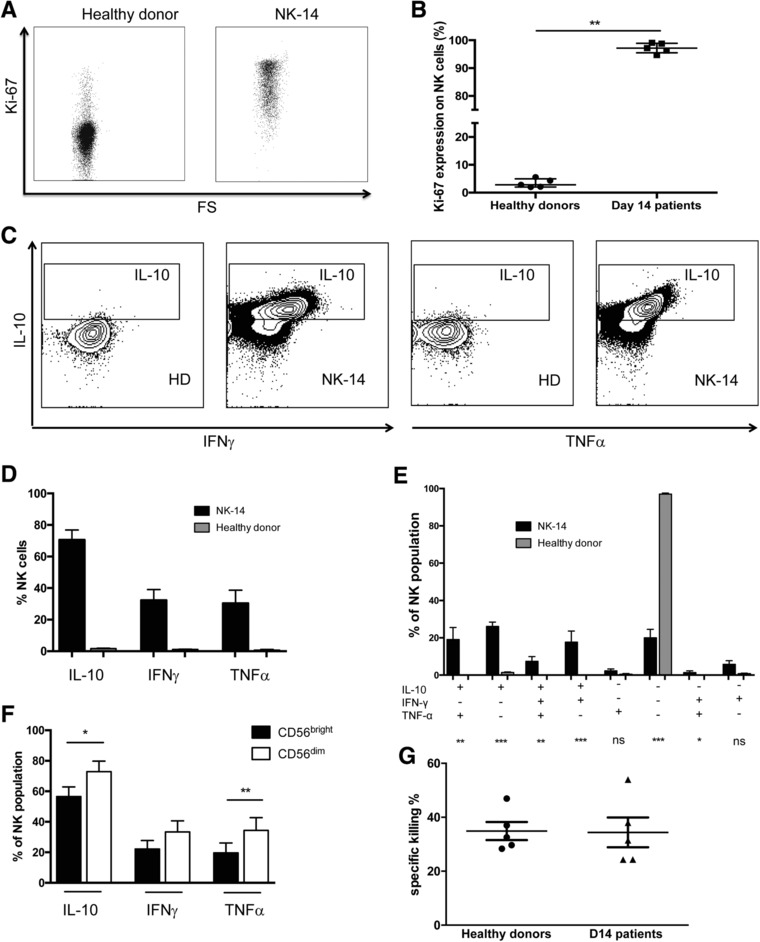
The functional profile of NK cells at day 14 after allo‐SCT. NK cells were enriched from freshly isolated PBMCs using the EasySepTM Human NK Cell Enrichment Kit (STEMCELL Technologies). Purified NK cells were analyzed by micro‐satellite analysis at the West Midlands Regional Genetics Laboratory to assess chimerism status and by flow cytometry. (A) Example plot of KI‐67 staining in one NK‐14 and NK cells from one healthy donor. (B) Comparison of Ki‐67 expression in NK‐14 (*n* = 5) and NK cells from HD (*n* = 5). (C) Example plot of intracellular staining of TNFα, IFNγ and IL‐10 from NK‐14 and NK cells from healthy donors without stimulation. (D) Comparison of cytokines production in NK‐14 and NK cells from healthy donors without stimulation. (E) Multiple cytokines production in NK‐14 and NK cells from healthy donors. (F) Comparison of cytokines production between CD56^bright^ and CD56^dim^ NK‐14 cell subsets. For all cytokine experiments: NK‐14 (*n* = 11) and healthy donors (*n* = 8). Data are pooled from 8 to 11 independent patients staining. (G) The cytotoxic activity of NK‐14 (*n* = 5) and NK cells from healthy donors (*n* = 5) was studied against K562 target cells at ratio 0.5:1. For all graphs the mean and standard error of the mean is depicted. ^*^ *p* < 0.05, ^**^ *p* < 0.01, ^***^ *p* < 0.005, two‐tailed, Mann–Whitney test for unpaired analyses, Wilcoxon matched‐pairs signed rank test for paired analyses between CD56^bright^ and CD56^dim^ NK cell subsets.

We next went on to investigate the origin and phenotype of NK‐14 cells in more detail. Microsatellite chimerism analysis was performed on purified CD3^−^CD56^+^ cells taken at day 14 and found NK‐14 cells were derived exclusively from the transplant donor (*n* = 3, data not shown). Given the remarkably high rate of NK cell reconstitution we then determined the proliferative status of the NK‐14 cells through the use of Ki67 expression (Fig. 3A). Although Ki67 was expressed in only 2.8% of NK cells within healthy donors, virtually all NK‐14 cells expressed Ki67 (Fig. 3B), reflecting an intense pattern of NK cell proliferation in the early post‐transplant period.

### NK‐14 cells demonstrate high levels of cytokine production and retain cytotoxic function

To study the functional properties of NK‐14 cells, we investigated their profile of intracellular cytokine production and cytotoxic potential in comparison to NK cells from healthy donors. Intracellular staining for cytokine production was carried out initially without prior stimulation of cells to assess baseline cytokine production in cells analysed directly ex vivo. As anticipated, NK cells taken from healthy donors displayed very low levels of cytokine production in the absence of mitogenic stimulation. Specifically, IL‐10, IFN‐γand TNFα production was seen in 1.5, 1.0 and 0.6% of cells respectively. However, NK‐14 cells were producing very high levels of cytokines in vivo, measurable directly in an ex vivo assay without mitogens. In particular, 71% of NK cells produced IL‐10, (*p* < 0.0001 versus healthy donors), 32% produced IFN‐γ (*p* < 0.0001) and 30% produced TNF‐α (*p* < 0.0001) (Fig. 3C and D). The serum concentration of IL‐10 at day 14 was also around 4 fold higher compared to values within healthy controls although no correlation was observed between this value and the incidence of GvHD (Supporting Information Fig. 3).

We further examined the combinatorial profile of cytokine production within NK‐14 cells. The most common profile was that of isolated IL‐10 production, seen in 26% of cells, followed by combined IL‐10 and TNF‐α or IL‐10 and IFN‐γ synthesis in 19 and 18% of cells respectively. A further 7% of NK‐14 cells were shown to be spontaneously producing all three cytokines while no cytokine production was seen in 20% of cells (Fig. 3E). Of note, only 9% of cells showed evidence of TNF‐α or IFN‐γ production in the absence of IL‐10, indicating that an immunoregulatory profile is dominant within NK‐14 cells. Cytokine production is generally considered to be feature of CD56^bright^ rather than CD56^dim^ NK cells and we therefore examined the pattern of expression in these subsets in NK‐14 cells. Interestingly, this pattern was reversed and higher levels of all three cytokines were observed within the CD56^dim^ subset (Fig. 3F).

NK‐14 cells were enriched through the use of magnetic negative selection and their cytotoxic activity against K562 target cells was then determined using a flow cytometry‐based cytotoxicity assay. The 38% of target cells were lysed by NK cells from healthy donors compared to 37% of cells when exposed to NK‐14 populations (Fig. 3G). As such, NK‐14 cells retain comparable levels of cytotoxic potential compared to NK cells from normal donors.

### Transcriptional activity within NK cells is markedly downregulated at day 14

**Figure 4:**
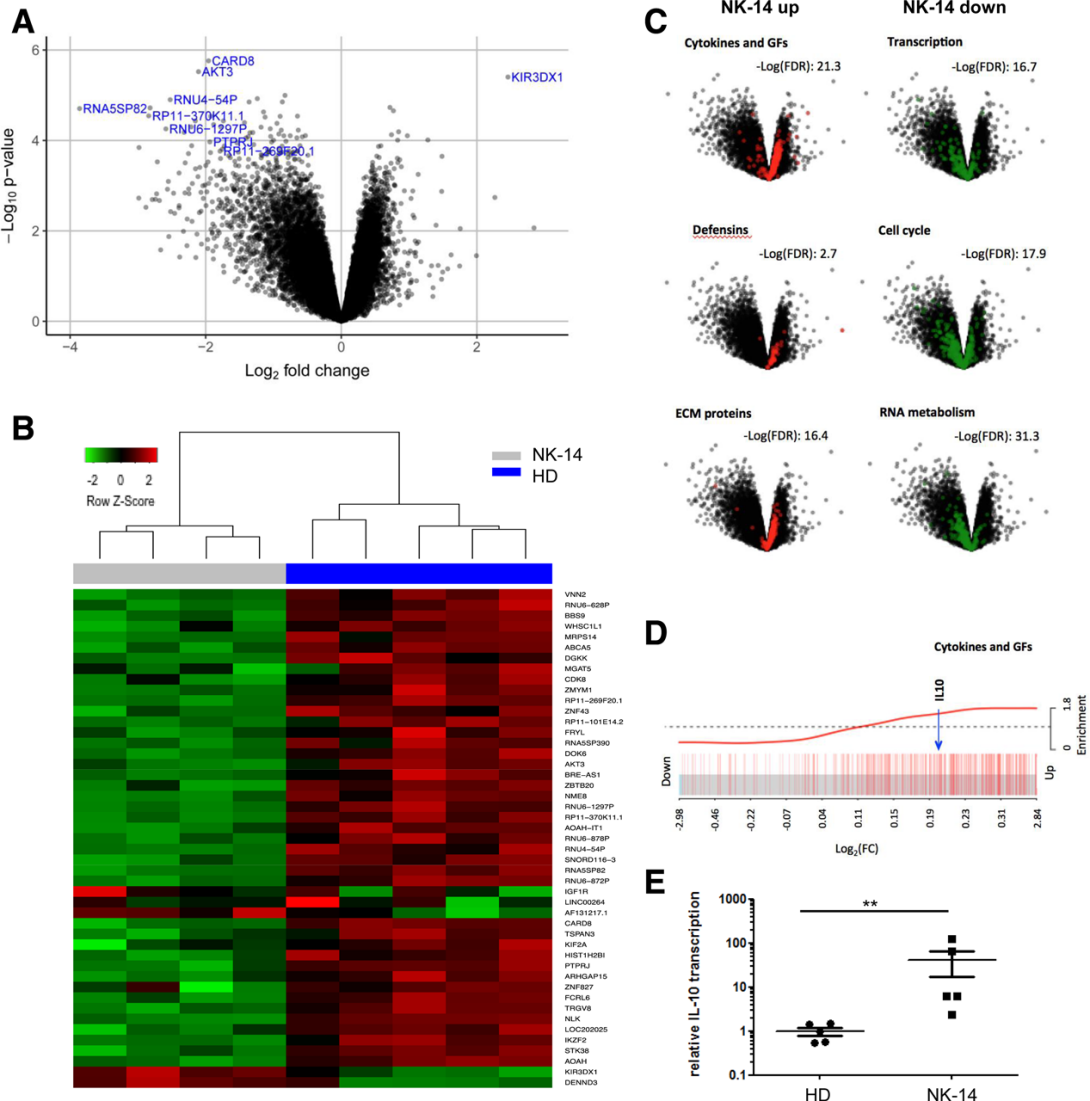
The transcriptional profile of NK cells at day 14 following allo‐SCT compared to healthy donor NK cells. (A) The NK cells were enriched, total RNA was extracted, labelled and hybridized to GeneChip® Human Transcriptome array 2.0 (Affymetrix, USA). Volcano plot demonstrating overall transcript profile of D14‐NK (*n* = 4) compared to NK cells from healthy donors (*n* = 5). The expression of genes shown to the left is reduced in NK‐14 and those to the right are increased. (B) Heatmap displaying the differentially expressed genes between D14‐NK and NK cells from healthy donors (absolute log_2_ FC > 1 and adjusted *p*‐value of < 0.1). (C) Gene set enrichment analysis shows that the genes for cytokines and growth factors, defensins and extracellular matrix proteins were specifically enriched in the upregulated transcripts, whilst genes involved in transcription, cell cycle and RNA metabolism were enriched in the downregulated genes. (D) A barcode plot highlighting enrichment of cytokines and growth factor genes, including IL‐10 within the upregulated transcripts. (E) The NK cells were enriched, total RNA was extracted and the transcription of IL‐10 (normalized by GAPDH transcription level) was studied with D14‐NK (n‐5) and NK cells from HD (*n* = 5) through qRT‐PCR. Data are pooled from three independent experiments. PCRs performed in five different donors. Data are represented as mean and error bars refer to standard error. The difference between was analyzed by Mann–Whitney test, with ^**^ *P* < 0.01.

We next examined the transcriptional profile of NK‐14 cells (*n* = 4) compared to NK cells from healthy donors (*n* = 5) to study the functional profile of these cells further. Most transcripts were expressed at a lower level in NK‐14 cells compared with healthy donors (Fig. 4A). Figure 4B displays differentially expressed genes which demonstrate absolute log fold change > 1 and for which the adjusted *p*‐value is < 0.1 (full gene list in Supporting Information Table 1). Gene set enrichment analysis demonstrated that genes involved in transcription, cell cycle and RNA metabolism were specifically enriched in the downregulated portion of the transcriptome (–Log(FDR) 16.7, 17.9 and 31.3 respectively) (Fig. 4C right panel). There was also significant downregulation of gene sets involved in NK cytotoxicity, the IFN‐γ pathway and NK‐related transcription factors (Supporting Information Table 2). The most significantly downmodulated genes included *CARD8*, which regulates caspase activation, and the serine/threonine protein kinase *AKT3*.

However, although the transcriptional profile was generally downregulated, genes for cytokines and growth factors, defensins and extracellular matrix proteins were enriched within the upregulated transcripts (‐Log(FDR) 21.3, 2.7 and 16.4 respectively) (Fig. 4C left panel). IL‐10 gene transcripts are upregulated in the NK‐14 cells by a factor of around 1.2 fold compared to NK cells from healthy donors and the specific position of IL‐10 within the upregulated cytokine and growth factor transcripts is shown in a barcode plot (Fig. 4D). qRT‐PCR data confirmed the upregulation of IL‐10 transcription in NK‐14 cells compared with healthy donors (Fig. 4E). The most significantly upregulated gene is KIR3DX1 (5.5‐fold in NK‐14 cells compared to HD) which is a killer cell immunoglobulin‐like receptor with unknown function.

### NK‐14 cell number predicts the risk of acute GVHD and overall survival

**Figure 5:**
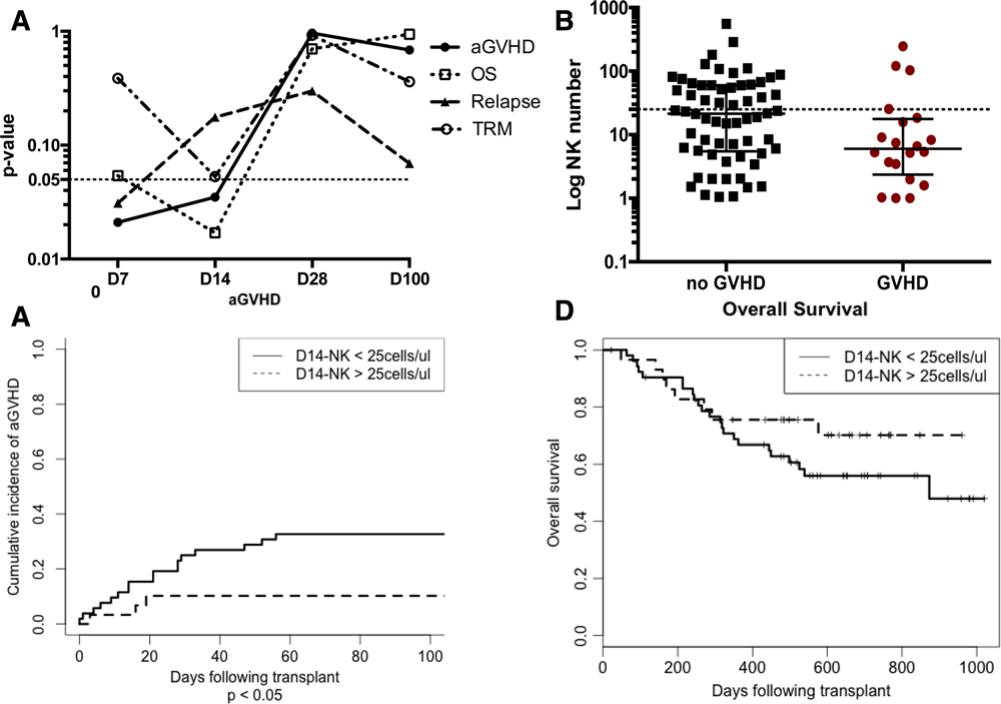
The magnitude of NK cell reconstitution in the first two weeks following allo‐SCT is predictive for the subsequent risk of acute GVHD and OS. (A) Receiver operating curves were plotted to assess the absolute NK cell count at various time points following SCT and its ability to discriminate between the clinical end‐points of: aGVHD, OS, relapse and transplant related mortality. A *p*‐value < 0.05 was used as the threshold for statistical significance (dash line). The *p* value (Y axis) versus NK number at different time points (D7, D14, D28, D100) (X axis) were plotted to demonstrate the association of NK number with different clinical outcomes. (B) Scatter plot (Mann‐Whitney test) to compare the NK number in the patients who developed acute GVHD and who did not develop acute GVHD. Dash line indicates the NK cell count of 25 cells/μl at day 14. (C) Cumulative incidence curve (Fine and Gray test) to compare the incidence of acute GVHD between different D14‐NK cell number group (cut‐off point as 25 cell/μL). *P* < 0.05. There are 52 patients in the group with NK cell number< 25 cell/μL and 30 patients in the group with NK cell number > 25 cell/μL. (D) Kaplan‐Meier curve (log‐rank test) to compare the overall survival between different D14‐NK cell number group (cut‐off point as 25 cell/μL).

Given the extreme numerical dominance of NK cells over T cells in the early post‐transplant period we then investigated if the reconstitution of NK cells was related to the clinical outcome of the procedure. We first assessed whether the number of NK cells at day 7, 14, 28 and 100 days post‐transplant could discriminate the subsequent risk of acute GVHD, transplant‐related mortality, relapse rate and overall survival. Receiver operating curves were plotted to assess the absolute NK cell count at various time points following SCT and its ability to discriminate between the clinical end‐points of: aGVHD, OS, relapse and transplant related mortality. A *p*‐value< 0.05 was used as the threshold for statistical significance. A significant association was seen between the number of NK cell at day 7 and 14 and acute GVHD, and with the NK number at day 14 and reduced overall survival. A higher rate of relapse was associated with lower number of NK cells at day 7 but this effect was lost by day 14 (Fig. 5A).

We went on to investigate the association between NK cell number and acute GVHD in more detail. Receiver operating characteristic (ROC) curve analysis demonstrated that the absolute NK cell count at D7 and D14 could discriminate the risk of acute GVHD with an area under curve (AUC) value of 0.68 (*p* < 0.05; 95% CI 0.54–0.82) and 0.66 (*p* < 0.05; 95% CI 0.51–0.80) respectively (Supporting Information Fig. 5). In addition to the total NK cell count, the absolute counts of several NK cell subsets were also predictive at these early time points (D7: CD56^bright^, CD56^dim^, NKG2A^+^, NKG2C^+^, KIR^+^, CD57^+^ and CD16^+^; D14: CD56^dim^, NKG2A^+^ and NKG2C^+^(Supporting Information Table 3)) but no single subset was uniquely associated.

### NK‐14 < 25 cells/μL is an independent predictor of acute GVHD in multivariate analyses

As we have shown the significant association between the number of NK cell at day 14 and acute GVHD, the NK‐14 number between the patients who developed acute GVHD and who did not develop acute GVHD were compared. The median NK number in the patients who developed acute GVHD was 5 cells/μL compared with 20 cells/μL in those who did not develop acute GVHD (Fig. 5B).

Using the ROC curve, we were able to appreciate how changing the cut off NK‐14 cell number used affected the sensitivity and specificity of the predictive test. We chose a cut off NK‐14 count of 25 cells/uL for univariate and multivariate analysis as this provided a high sensitivity (85%) for predicting acute GVHD with a specificity of 44%. In multivariate analysis, NK cell number of < 25 cells/μL, HLA‐mismatch and conditioning intensity remained independently associated with an increased risk of acute GVHD (*p* = 3.5 × 10^−2^, *p* = 5.1 × 10^−6^ and *p* = 9.1 × 10^−4^ respectively) (Table 3 and Fig. 5C). Patients who achieved this cell count at day 14 also showed a trend towards increased survival but this was not significant (Fig. 5D). An NK‐14 cell count of > 25 cells/μL was not associated with any differences in chronic GVHD, disease relapse, relapse mortality or transplant‐related mortality (Supporting Information Fig. 6A 6B 6C and 6D).

**Table 3:** Factors predicting development of acute GVHD (grades II–IV)

		HR	95% CI	*P*‐value
**Univariate analysis**				
Age	< 50	1		
	≥ 50	0.496	0.208–1.18	0.11
Sex	Male	1		
	Female	0.976	0.392‐2.43	0.96
Diagnosis	myeloid	1		
	lymphoid	1.53	0.639‐3.65	0.34
	marrow failure	0.734	0.112‐4.8	0.75
Sorror score	0	1		
	≥ 1	2.11	0.856‐5.19	0.1
DRI	low‐int	1		
	high‐v high	1.37	0.576‐3.27	0.48
Intensity	RIC	1		
	MA	4.24	1.81‐9.96	**9.1 × 10^−4^**
CMV at risk	Yes	0.888	0.3‐2.62	0.83
	No	1		
Male female mismatch	Yes	1.79	0.726‐4.41	0.21
	No	1		
Donor	Unrelated	1.99	0.676‐5.86	0.21
	Sibling	1		
TCD	Campath	1		
	ATG	1.07	0.34‐3.4	0.9
HLA	0	1		
mismatch	≥ 1	4.41	1.81‐10.7	**0.0011**
D14‐NK	< 25	3.52	1.02‐12.1	**0.046**
	≥ 25	1		
**Multivariate analysis**				
Conditioning	RIC	1		
	MA	6.759	2.19‐20.9	**9.1 × 10^−4^**
HLA mismatch	0	1		
	≥ 1	16.495	4.94‐55.0	**5.1 × 10^−6^**
D14‐NK	< 25	5.81	1.13‐29.8	**3.5 × 10^−2^**
	≥ 25	1		

The Fine and Gray model for subdistributional hazard based on the cumulative incidence function was used with death as a competing risk for development of aGVHD. A landmark approach was required with time from day 14 to development of aGVHD used for analysis. DRI, disease risk index; RIC, reduced intensity conditioning; MA, myeloablative; TCD, T cell depletion.

Using a lower NK‐14 cut off of 5 cells/μL increases the specificity of the predictive test, although there is a reciprocal decrease in the sensitivity. Interestingly, we found that patients with an NK‐14 cell count of < 5 cells/uL demonstrated significantly reduced overall survival (Supporting Information Fig. 6E) on both univariate and multivariate analysis, indicating that a relatively low level of NK reconstitution is sufficient to deliver a survival advantage with incremental improvements in NK recovery associated with additional improvements in patient outcome following allo‐SCT.

## Discussion

Immunological processes determine the majority of the clinical features of allo‐SCT but the mechanisms of immune regulation after transplant are poorly understood despite 40 years of clinical practice 17. The rapid reconstitution of NK cells is a characteristic feature of allo‐SCT 18 and whilst donor T cells are crucial in the alloreactive immune response 19, 20 there is growing appreciation of the importance of NK cells in determining clinical outcome 21. We focussed our studies within the first two weeks after transplantation as this early phase of immune reconstitution has been relatively poorly investigated and yet plays a critical role in determining the profile of alloreactive immunity and clinical outcome.

Our findings confirm that NK cells recover rapidly following T cell depleted allo‐SCT 9, 10. This expansion, at a time when T cell numbers are highly suppressed, leads to a profound alteration in the ratio of NK and T cells within peripheral blood. In healthy individuals the NK:T cell ratio is approximately 0.17, whereas at day 14, NK cells outnumber T cells by 40 to 1, representing a greater than 200 fold change in NK:T cell ratio. It should be noted that the transplant conditioning regimens in our study incorporated T cell depletion as a strategy to reduce the risk of GVHD 22, 23.

We identified a biphasic pattern of reconstitution within NK cell subsets. There was an initial slight increase in the proportion of CD56^dim^ NK cells at day 14 and subsequent expansion of CD56^bright^ NK cells, to 31% of the NK cell population by day 100. The expansion of mature CD56^dim^ NK cells likely represents homeostatic proliferation of donor NK cells adoptively transferred within the stem cell product whereas the subsequent increase of the less mature CD56^bright^ NK cell population probably reflects de novo production of NK cells from donor stem cells 11.

Remarkably, almost all NK‐14 cells were undergoing proliferation, compared to less than 3% of healthy donor cells. This reflects intense homeostatic proliferation and has been observed in lymphopenic mouse models 24, 25. IL‐15 is critical for NK proliferation and survival and, as serum IL‐15 levels rise in the first two weeks following allo‐SCT, is also likely to represent an important mechanism following transplantation 26.

NK‐14 cells display increased expression of NKG2A and lower levels of CD57 on the CD56^dim^ subset. This phenotypic profile has similarities with cytokine‐induced memory‐like (CIML) NK cells, which are induced in vitro by IL‐12, IL‐15 and IL‐18 stimulation 27. Allo‐SCT conditioning is associated with high cytokine levels, sometimes termed the ‘cytokine storm’, and NK‐14 may represent the in vivo correlate of CIML 28. Functional similarities include vigorous cytokine production. CIML NK cells are potent anti‐leukaemia effector cells 29 and it is possible that NK‐14 play an important role in the GvL response as we also demonstrated that D14‐NK cells retain levels of cytotoxic activity that are comparable to healthy donors.

NK‐14 cells express increased levels of chemokine receptors, including CCR7 which guides trafficking to lymph nodes 30, CXCR4 which mediates bone marrow homing 31 and CCR9 which directs cells towards the thymus and gastrointestinal tract 32. Overall, NK‐14 cells acquire the ability to enter a range of lymphoid and peripheral tissues.

Perhaps the most striking feature of NK‐14 cells was the pattern of intense and spontaneous cytokine production. This profile was observed without mitogenic stimulation in vitro, was not demonstrable in cells isolated from healthy donor and has been noted in murine models during homeostatic proliferation 33. IL‐10 was the dominant cytokine, being expressed in 70% of NK‐14 cells in comparison to IFN‐γ or TNF‐α expression in less than half of this proportion. Interestingly, NK cells isolated at day 28 following HSCT continued to demonstrate spontaneous production of IL‐10 within 0.34% of cells, compared to 0.06% of healthy donors (*p* < 0.05), although this does demonstrate a relatively short timecourse of IL‐10 production in the post‐transplant period (Supporting Information Fig. 4). This pattern has similarities with murine models studied by Tarrio and colleagues 34. In these models, homeostatic proliferation leads to epigenetic reprogramming of the IL‐10 locus and a switch to IL‐10 production in a phenomenon termed ‘proliferation‐dependent conditioning’ of NK cells. This switch from an inflammatory to regulatory phenotype utilizes the high frequency of NK cells to apply negative pressure on the adaptive immune system. IL‐10 production by proliferating human NK cells has also been demonstrated in vitro following incubation with both IL‐2 and IL‐12 35.

IL‐10 is an anti‐inflammatory mediator that is vital in limiting immunopathology 35, 36, 37 and can also suppress NK‐mediated licensing of DC activation 38 and modulate NK deletion of DCs 39. Our finding that NK cells strongly outnumber T cells, express high levels of IL‐10 and can migrate to secondary lymphoid tissue together indicate that they may suppress the generation of the T cell mediated alloreactive immune response. Furthermore, immunoregulatory NK cells within peripheral tissues may serve to limit tissue damage mediated by alloreactive effector cells. The ability of NK cells to directly kill antigen presenting cells (DCs) or activated T cells may also play an important role 21, 40, 41, 42.

Allo‐SCT is a therapeutic intervention and is profoundly different to physiological or pathophysiological processes encountered during evolution. However, IL‐10 production within NK cells has been demonstrated following systemic infections and the induction of IL‐10 production in NK‐14 cells may represent an extension of the physiological response to regulate inflammation following episodes of moderate lymphopenia during infection 43, 44. Although our study is focused on patients undergoing allo‐SCT, our findings provide further insight into the normal physiological function of NK cells.

Microarray analysis demonstrated a predominant profile of ‘transcriptional exhaustion’ within NK‐14 cells, with marked downregulation in the expression of most genes. Human NK cells have a turnover time of around 2 weeks within blood 45 and thus many cells will be undergoing senescence following 14 days of intense activation and proliferation. However, genes associated with cytokines and growth factors, defensins and extracellular matrix proteins were selectively enriched within upregulated transcripts, corroborating our findings that NK‐14 cells are functional cytotoxic cells demonstrating prolific cytokine production. The mRNA level of the KIR gene *KIR3DX* 46 was increased by 5.5 fold in NK cells at day 14 and whilst the function of KIR3DX1 is unknown.

Our study investigated whether the temporal profile of NK cell reconstitution correlated with clinical outcome. The association between NK cell reconstitution and acute GVHD and overall survival was seen only with NK cell number at day 7 and day 14, and was subsequently lost when the NK count was determined at day 28 and day 100. Therefore, the expansion and function of the mature donor NK cells infused with the stem cell graft play a dominant role in determining transplant success. The whole NK‐14 cell population was implicated, as we did not identify a specific subset of NK cells responsible for determining the relationship with clinical outcome. Although it is intuitive to expect that the NK‐14 cell count is dependent on the absolute number of NK cells infused within the stem cell graft, this correlation was not statistically significant (data not shown). Therefore, factors present within the patient, such as cytokine levels, maybe more influential in determining whether a patient reconstitutes sufficient NK cells to provide protection from adverse outcome. Importantly, lower NK‐14 cell numbers were not associated with an increased risk of relapse, indicating retention of the GVL response and consistent with other studies demonstrating that NK cells can dissociate GVHD and GVL 12, 15.

**Figure 6:**
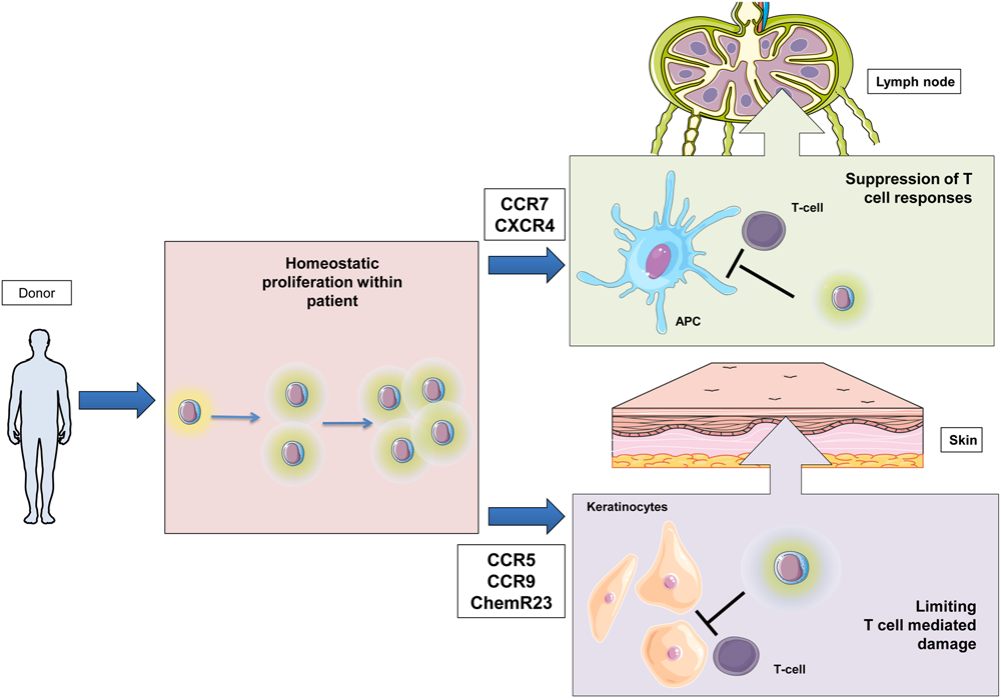
Diagram to show donor NK cells that produce IL‐10 in the early post transplant period suppress the development of acute GVHD in patients undergoing allo‐SCT. Mature NK cells are transferred from the donor to the patient at the time of stem cell infusion and undergo intense homeostatic proliferation and a switch to an immunoregulatory phenotype with dominant spontaneous production of IL‐10. These NK cells express chemokine receptors required for migration to secondary lymphoid tissues, where they are likely to play an important role in suppressing the development of alloreactive T cells, or to peripheral tissues where they may limit T cell damage. (This figure has been produced using resources from Servier Medical Art).

In conclusion, higher numbers of NK‐14 cells are strongly correlated with a reduced risk of acute GVHD and our finding that such cells express high levels of IL‐10, and are targeted to lymphoid and peripheral tissue, provides a direct mechanism for this association (Fig. 6). NK cells may traffic to the lymph node, where IL‐10 could suppress the generation of alloreactive T cell responses, or their effect may be mediated by suppression of alloreactive T cells within peripheral tissue. These findings have relevance for clinical practice. NK‐14 cell number may be a biomarker for clinical outcome, particularly when combined with additional measures such as serum analysis 47 or T cell count 48, 49. Furthermore, optimisation of early donor NK cell engraftment may impact on patient outcome, possibly through graft engineering or by manipulation of the cytokine microenvironment.

## Materials and methods

### Patients and sample collection

NK cell reconstitution was examined in a cohort of 82 consecutive patients who underwent allo‐SCT using mobilised peripheral blood. Patients received either alemtuzumab (10 mg/kg × 5 days) or anti‐thymocyte globulin (ATG) prior to stem cell infusion as in vivo T cell depletion. The clinical characteristics and transplant details for patients in the study are summarised in Table 1. RIC and myeloablative conditioning regimes were defined using published guidelines 50. HLA typing was determined at HLA‐A, ‐B, ‐C, ‐DRB1 and –DQB1 using high‐resolution techniques. Unrelated donors were either HLA matched (10/10) or mismatched at one loci (9/10).

In addition to in vivo T cell depletion, patients received ciclosporin +/‐ methotrexate for GVHD prophylaxis according to local protocols. Ciclosporin was commenced pre‐transplant, dose‐adjusted to achieve therapeutic levels and tapered from D+90 in the absence of GVHD. The diagnosis and grading of acute GVHD was carried out in accordance with consensus criteria 51.

Clinical data and samples were obtained after written informed consent in accordance with the Declaration of Helsinki and approved by the South Birmingham Research Ethics Committee (Q5/Q2707/175). Blood samples were taken 1 week pre‐transplantation and then at day 7 (range 5–8 days), day 14 (range 12–15 days), day 28 (range 3–5 weeks) and day 100 (range 3–4 months) post transplant.

### Flow cytometry: surface marker phenotype, chemokine receptor profile, cytokine production and cell number estimation

The experiments with T cells in the study always followed the MIATA (Minimal Information About T cell Assays) guidelines.

PBMCs were isolated by density centrifugation. Mononuclear cells were incubated with surface antibodies on ice for 20 minutes with Propidium iodide (PI) as viability dye. The antibodies for the surface phenotype staining include Qdot655 conjugated anti‐CD3 (clone S4.1, Invitrogen); APC‐Cy7 conjugated anti‐CD56 (clone HCD56, Biolegend); V500 conjugated anti‐CD16 (clone 3G8, BD Biosciences); Pacific Blue conjugated anti‐CD57 (clone HCD57, Biolegend); PC5.5 conjugated anti‐CD158a (clone EB6, Beckman Coulter); PC7 conjugated anti‐CD158b (clone GL18, Beckman Coulter); AF700 conjugated anti‐CD158e (clone DX9, Biolegend); APC conjugated anti‐NKG2A (clone Z199, Beckman Coulter); PE conjugated anti‐NKG2C (clone 134591, R & D systems).

The chemokine receptor staining included two panels. The same antibodies (anti‐ CD3, CD56 and CD16) as surface phenotype panel were used to gate NK cells. The chemokine receptor antibodies from the first panel include: PerCPCy5.5 conjugated anti‐CXCR3 (clone 1C6, BD Biosciences); APC conjugated anti‐CCR7 (clone 150503, R & D Systems); PeCy7 conjugated anti‐CCR5 (clone 2D7, BD Biosciences); FITC conjugated anti‐ChemR23 (clone BZ332, Bio‐Rad antibodies:formerly ABD serotec); AF700 anti‐CXCR4 (clone 12G5, R & D Systems); PE conjugated anti‐CCR6 (clone G034E3, Biolegend). The chemokine receptors antibodies from the second panel include: APC conjugated anti‐ CCR9 (clone 112509, R & D Systems) and PerCPCy5.5 conjugated anti‐CX3CR1 (clone 2A9‐1, Biolegend)

Ex vivo cytokine production determined using the intracellular antibodies panel, included: PE conjugated anti‐CD3 (clone UCHT1, Beckman Coulter); APC‐cy7 conjugated anti‐CD56 (clone HCD56, Biolegend); Pe‐Cy7 conjugated anti‐TNFα (clone MAb11 eBioscience);

AF700 conjugated anti‐IFNγ (clone 4S.B3, Biolegend); PE conjugated anti‐IL‐10 (clone JES3‐19F1, Biolegend).

Data was acquired on an LSR‐II flow cytometer and analysed using FACSDiva software.

For samples taken at day –7, day 28 and day 100, lymphocyte counts were available from clinical laboratory full blood counts. Samples from day 7 and day 14 were estimated using BD Trucount Tubes (BD Biosciences).

### Chimerism analysis

NK cells were enriched from freshly isolated PBMCs using the EasySepTM Human NK Cell Enrichment Kit (STEMCELL Technologies). Purified NK cells were analysed by micro‐satellite analysis at the West Midlands Regional Genetics Laboratory to assess chimerism status.

Ki‐67 assay NK cells were isolated as described above. Ki‐67 expression on the purified NK cell population was assessed using the FITC Mouse Anti‐Human Ki‐67 Set (BD Pharmingen).

### NK cell cytotoxicity assay

NK cells were purified using EasySepTM Human NK cell enrichment kit (STEMCELL Technologies) (purity of sorted NK cells was 90–95% of CD3 negative and CD56 positive lymphocytes) and activated. Meanwhile, K562 cells were stained with CFSE dye. Labelled K562 cells were either incubated with RPMI (negative control) or with activated NK cells at a E/T ratio of 0.5:1 for 16 h. Cells were subsequently extracted and a fixed volume analysed on the BD Accuri™ flow cytometer (BD Bioscience) to gain an absolute cell count.

% specific lysis was calculated by 100 × {1 − [(experimental group cell count)/(control cell count)]}. PI was used to gate the live populations.

### Gene expression profiling

Magnetically isolated NK cells were stained with anti‐CD3 PE, anti‐CD56 APC‐Cy7, and CD3‐ and CD56+ live cells were sorted with the Astrios cell sorter (BD Biosciences) as an added enrichment step. The purity of the sorted NK population was 99% of CD3 negative and CD56 positive lymphocytes. The sorted NK cell populations were sent to AROS Applied Biotechnology A/S (Aarhus N, Denmark) as dry cell pellets. Total RNA was extracted, labelled and hybridized to GeneChip® Human Transcriptome array 2.0 (Affymetrix, USA). Microarray data is available in the ArrayExpress database (www.ebi.ac.uk/arrayexpress) under accession number E‐MTAB‐5474. Raw data was processed using Affymetrix's Expression Console software using default RMA parameters. Statistical analysis of differential expression was performed using the R package limma with Benjamini‐Hochberg method applied to adjust *p*‐values for multiple testing. Gene‐set enrichment analysis was performed using the Bioconductor R package GAGE. Curated gene sets for canonical signaling pathways were obtained from the Molecular Signalling Database.

### Statistical analysis

Mann–Whitney's non‐parametric method was used to compare healthy donor NK and D14‐NK cells. The Wilcoxon matched pairs signed rank test was used to compare CD56bright and CD56dim NK cell subsets.

Receiver operating characteristic (ROC) curves were plotted to assess the prognostic relationship between NK cell reconstitution and grade II‐IV acute GVHD. The area under the curve (AUC) and 95% confidence intervals for the NK cell subsets and time points were compared and the NK cell population at day 14 (NK‐14) identified for further investigation.

A landmark approach was taken for all survival analyses, with time from day 14 to clinical event used in analysis. The cumulative incidence of grade II‐IV acute GVHD was assessed in combination with ‘death from any cause’ as a competing risk. Patients with NK‐14 < 25 NK cells/μL had an increased risk of acute GVHD (HR 3.52, 95% CI 1.02‐12.1, p = 0.046)(Fig. 5B). HLA mismatch ≥1 (HR: 4.41, 95% CI: 1.81–10.7, *p* = 0.0011) and myeloablative conditioning (HR: 4.24, 95% CI: 1.81–9.96, *p* = 9.1 × 10–4) also significantly increased the risk of acute GVHD on univariate analysis. The multivariate adjustment for factors predicting the cumulative incidence of grade II‐IV acute GVHD was performed within the framework of competing risks using the Fine and Gray method. Factors significant on univariate analysis were included in the model. A competing risk approach was also taken in assessing the effect on TRM (using death from relapse as the competing event) and RR (using TRM as the competing event).

To investigate the effect of D14‐NK cell reconstitution and clinical variables on overall survival, survival curves were plotted using the Kaplan‐Meier method and assessed with the log‐rank method. The Cox proportional hazards model was used for multivariate analyses.

All statistical tests were two‐sided and the level of statistical significance utilized was 0.05. Statistical analysis was carried out using R (version 3.2.1 GUI 1.66 Mavericks build (6956)), SPSS (IBM version 22) and Prism (GraphPad version 6.0b). For competing risk analysis, we used the cmprsk package and ccr‐addson function written for R (http://www.stat.unipg.it/luca/R).

## Conflict of interest

The authors declare no financial or commercial conflict of interest.

**Abbreviations**
Allo‐SCT: allogeneic stem cell transplantation
CMV: cytomegalovirus
GVHD: Graft versus host diseases
NK: natural killer

## Supporting information

Peer review correspondence

Supplementary Material
