# Supplementary material for: NK cells produce high levels of IL‐10 early after allogeneic stem cell transplantation and suppress development of acute GVHD
Source: Eur J Immunol. 2017 Oct 27;48(2):316–29. doi: 10.1002/eji.201747134 (PMC5836991; doi:10.1002/eji.201747134)
Supplement: Supplementary file 2 — Supplementary Material [file EJI-48-316-s002.pdf]

European Journal of Immunology

Supporting Information for DOI 10.1002/eji.201747134

Yuen Ling Tracey Chan, Jianmin Zuo, Charlotte Inman, Wayne Croft, Jusnara Begum, Joanne Croudace, Francesca Kinsella, Luke Maggs, Sandeep Nagra, Jane Nunnick, Ben Abbotts, Charles Craddock, Ram Malladi and Paul Moss

NK cells produce high levels of IL-10 early after allogeneic stem cell transplantation and suppress development of acute GVHD

© 2017 The Authors. *European Journal of Immunology* published by WILEY-VCH Verlag GmbH & Co.KGaA, Weinheim.

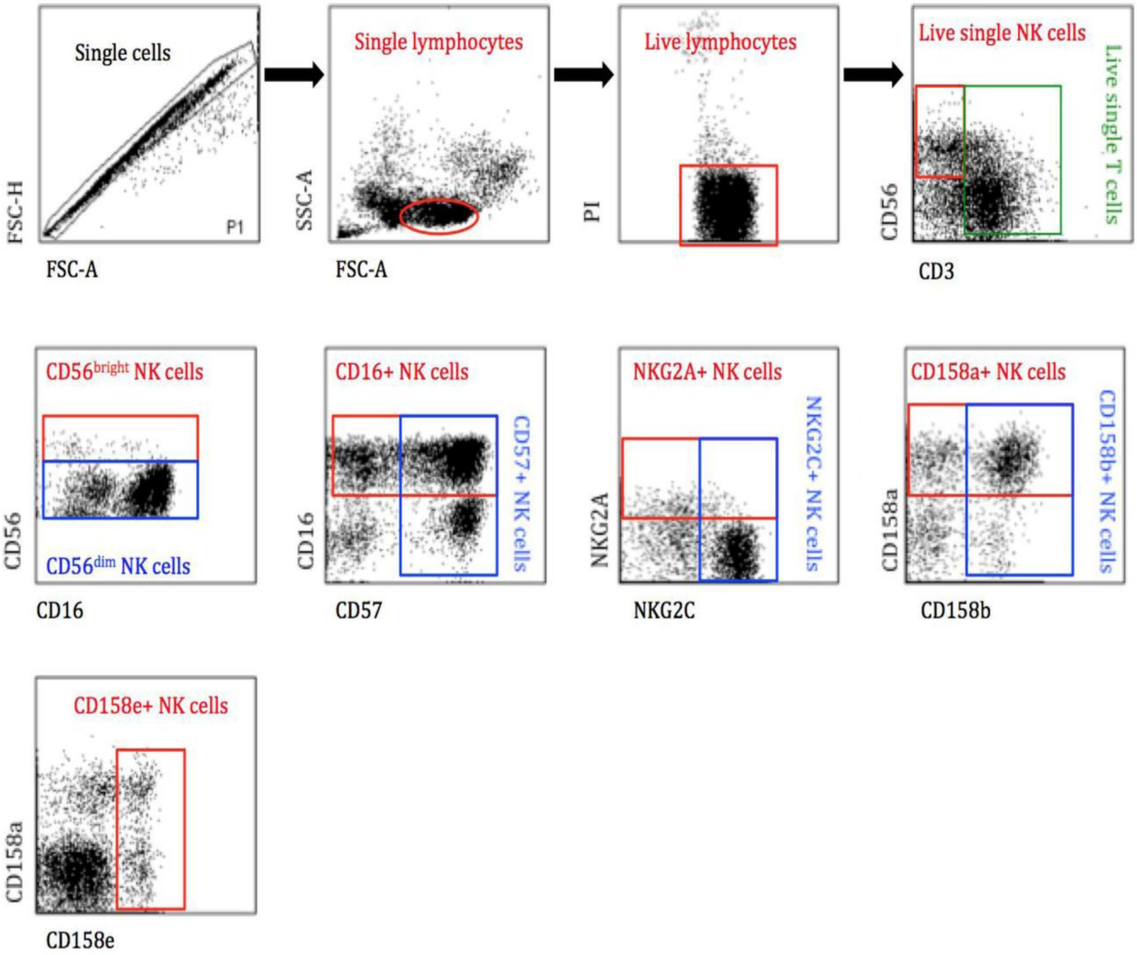

Supplementary Figure 1. Gating strategy to determine immunophenotype of NK cells and their subsets

The top four panels are gated down sequentially using the preceding panel's gate as the total population. The live single NK cell gate is used to populate the bottom five plots.

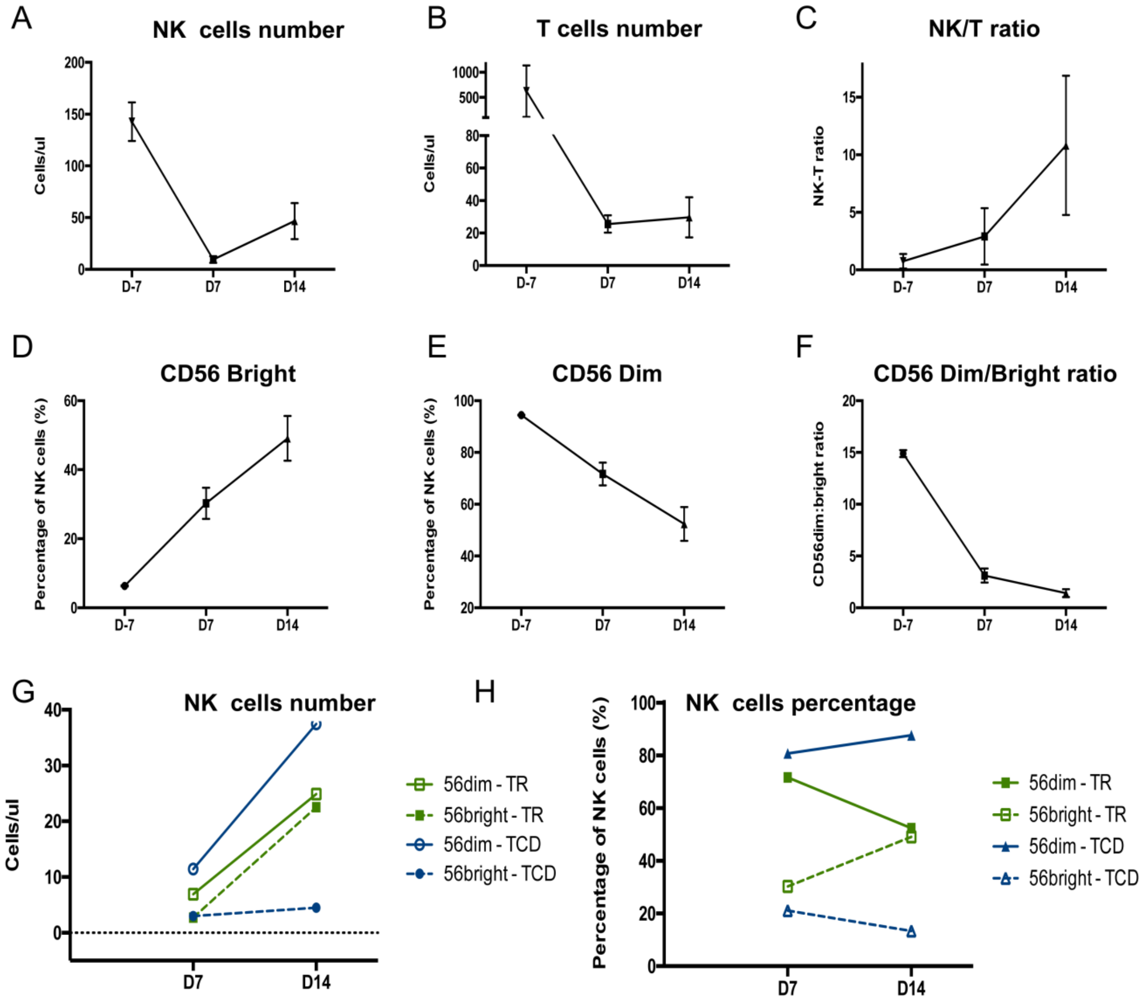

Supplementary Figure 2. NK and T cell reconstitution following T cell replete (TR) allo-SCT

(**A**) The reconstitution of the number of NK cells in the first 14 days following TR allo-SCT (**B**) The reconstitution of the number of T cells in the first 14 days following TR allo-SCT. (**C**) The ratio of NK cell: T cell number up to 14 days following TR allo-SCT. (**D**) CD56^{bright} NK cells and (**E**) CD56^{dim} NK cells expressed as a percentage of the total NK cell population in the first 14 days following TR allo-SCT. (**F**) The ratio of CD56^{dim} NK cells: CD56^{bright} NK cells plotted following TR allo-SCT. All graphs depict mean and error bars represent the standard error of the mean. Graphs display the mean with error bars representing the standard error. D -7 (n=2), D 7 (n=9), D 14 (n=8). (**G**) The comparison of the reconstitution of the number of CD56^{dim} NK cells and CD56^{bright} NK cells between T cell deplete and T cell replete allo-SCT. (**H**) The comparison of the percentage of CD56^{dim} NK cells and CD56^{bright} NK cells between T cell deplete and T cell replete allo-SCT..

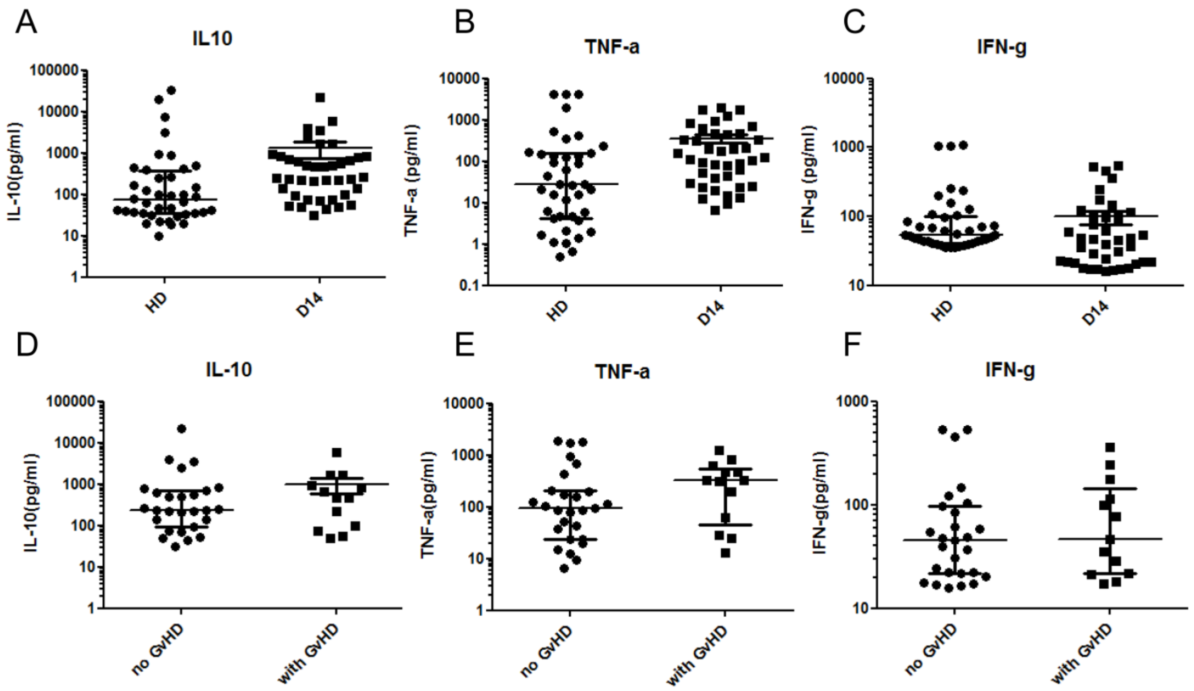

Supplementary Figure 3. The concentration of IL-10, TNF-α and IFN-γ in the serum from day 14 patients after allo-SCT.

The concentration of IL-10 **(A)**, TNF-α **(B)** and IFN-γ **(C)** are studied using ELISA (R&D system, DY217B, DY410 and DY485) with the serum from healthy donors (n=40) and day 14 patients after allo-SCT (n=40). The comparison of IL-10 **(D)**, TNF-α **(E)** and IFN-γ **(F)** between the day 14 patients who developed GvHD (n=13) and who did not develop GvHD (n=27).

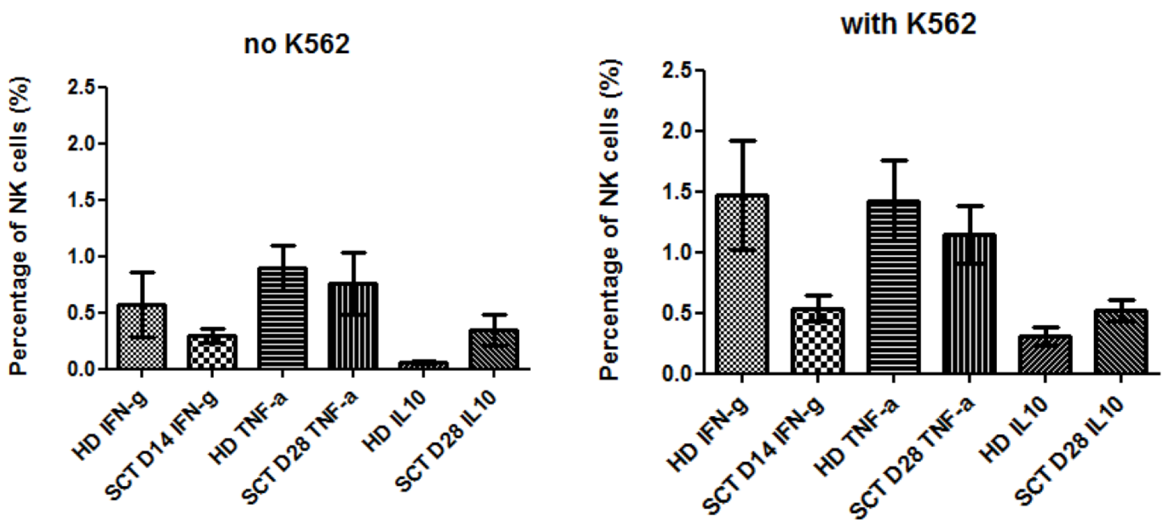

Supplementary Figure 4. The cytokines profile of NK cells of patients at day28 after allo-SCT. The whole PBMC were either stimulated with K562 as 1:1 ratio or with media only for 18hrs. Then the intracellular expression of IL-10, TNF-α and IFN-γ in NK cells (gated as CD3 negative and CD56 positive population) were stained from patients (n=5) at day28 after all-SCT. The data were shown as the percentage of NK cells producing cytokines without K562 stimulation **(A)** or with K562 stimulation **(B)**. The NK cells from healthy donors (n=5) were used as control.

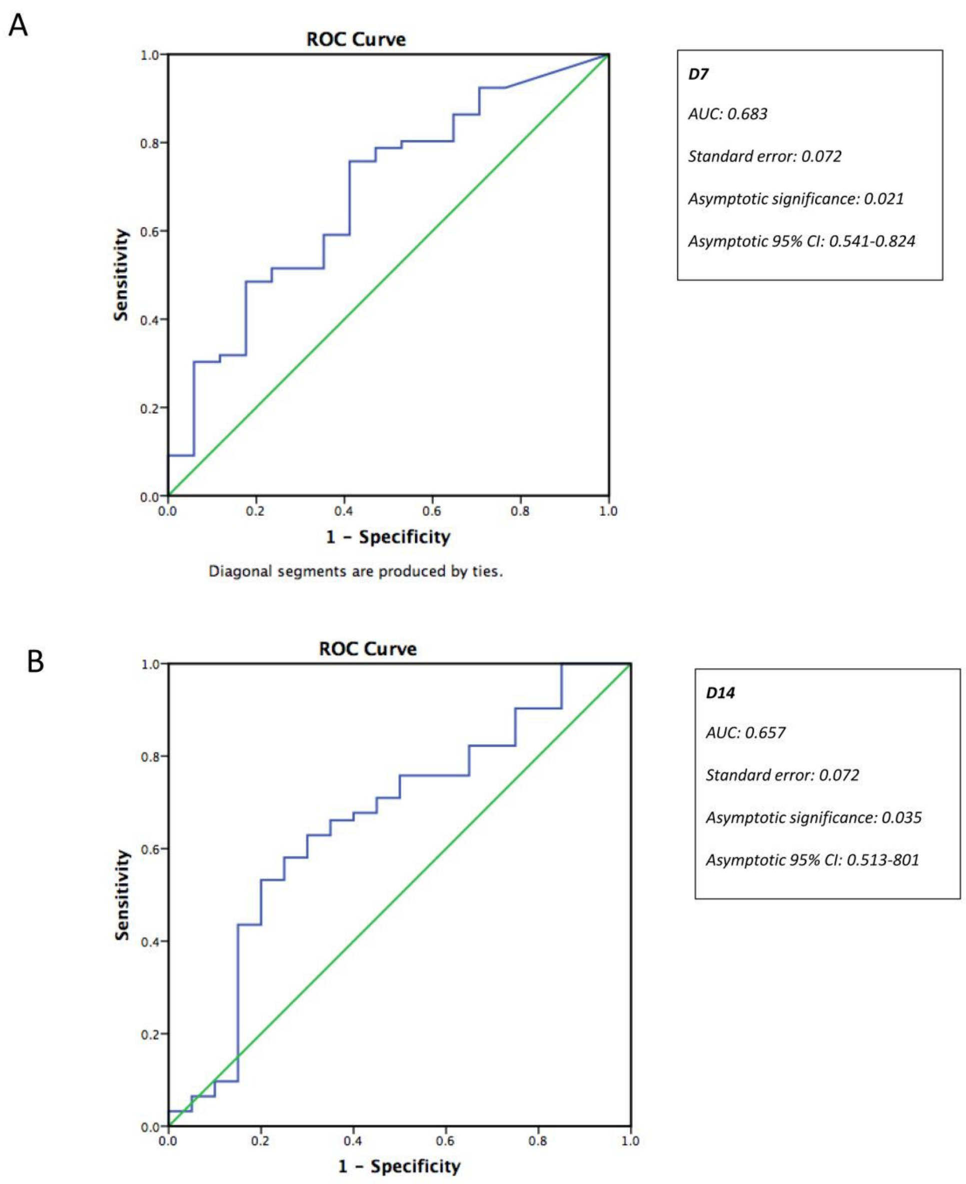

Supplementary Figure 5. Receiver operating characteristic curve analysis demonstrates the ability of the absolute NK cell count at D7 (A) and D14 (B) to predict aGVHD.

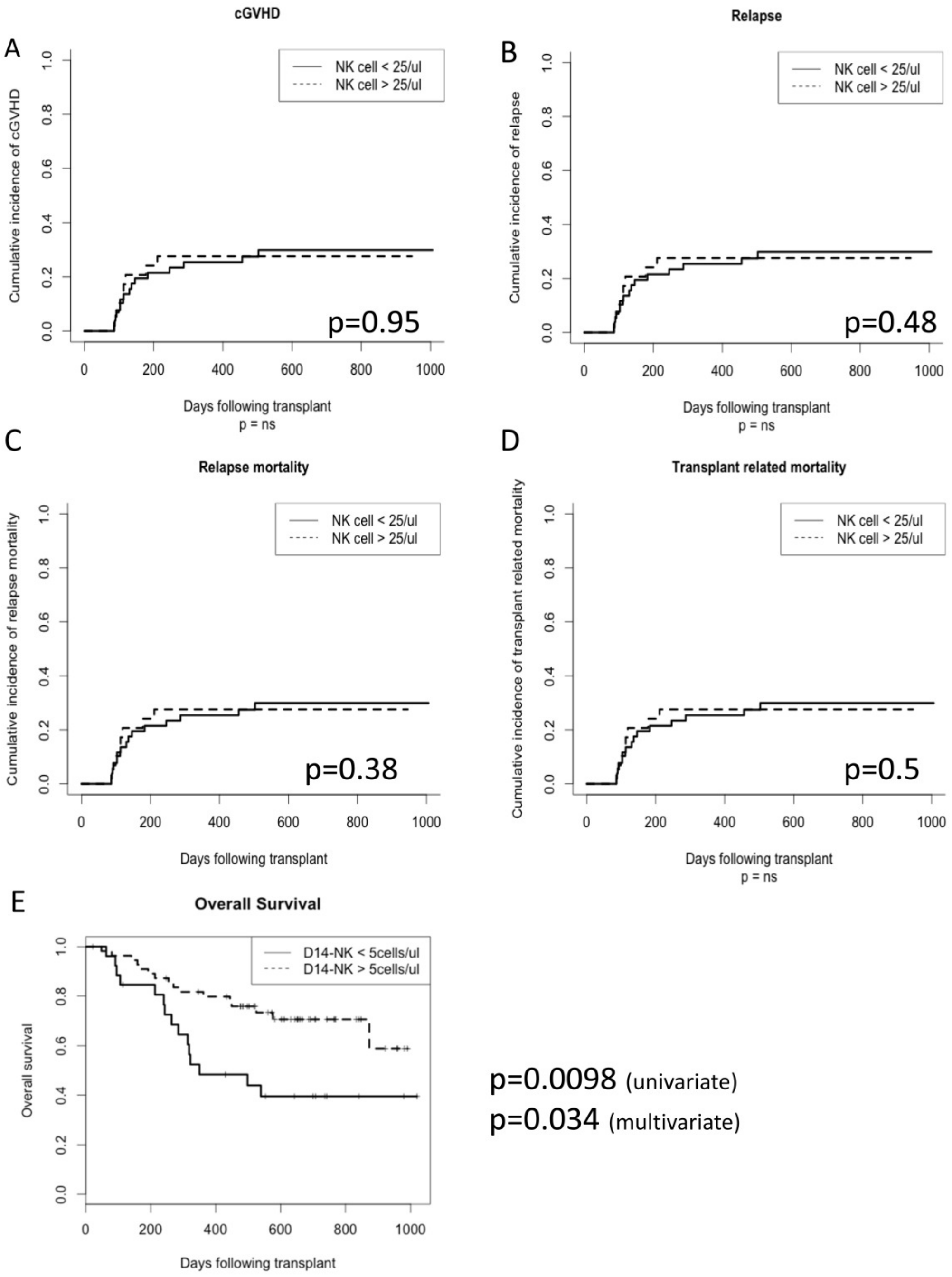

Supplementary Figure 6. D14-NK <25 cells/µl is not a significant predictor of the cumulative incidence of chronic GVHD, disease relapse, relapse mortality or transplant related mortality. An absolute NK-14 cell count <5cells/µl is associated with an increase in mortality. Cumulative incidence curve (Fine and Gray test) to compare the incidence of chronic GVHD (A), relapse (B), relapse mortality (C) and transplant related mortality (D) between different NK-14 cell number groups (cut-off point as 25 cell/µl). All p- values are for univariate analysis. (E) Kaplan-Meier curve (log-rank test) to compare the overall survival between different NK-14 cell number group (cut-off point 5 cell/µl).

Genes upregulated on NK-14 vs HD			
Gene Name	log_2FC	Adjusted *p*	Gene Description
KIR3DX1	2.46	0.05	killer cell immunoglobulin-like receptor, three domains, X1
DENND3	1.28	0.08	DENN/MADD domain containing 3
MIR4633	0.97	0.07	microRNA 4633
CTC-467M3.3	0.85	0.07	novel transcript
TPX2	0.83	0.08	TPX2, microtubule-associated
SNAR-I	0.77	0.06	small ILF3/NF90-associated RNA I
NUSAP1	0.74	0.07	nucleolar and spindle associated protein 1
RP11-388N2.1	0.72	0.06	putative novel transcript
NFKB2	0.58	0.09	nuclear factor of kappa light polypeptide gene enhancer in B-cells 2 (p49/p100)
FAM218A	0.55	0.09	family with sequence similarity 218, member A
Genes downregulated on NK-14 vs HD			
RNA5SP82	-3.86	0.06	RNA, 5S ribosomal pseudogene 82
SNORD116-3	-2.98	0.09	small nucleolar RNA, C/D box 116-3
RP11-370K11.1	-2.84	0.06	novel transcript
RNU6-872P	-2.82	0.06	RNA, U6 small nuclear 872, pseudogene
RNU6-1297P	-2.59	0.06	RNA, U6 small nuclear 1297, pseudogene
RNU4-54P	-2.52	0.06	RNA, U4 small nuclear 54, pseudogene
BRE-AS1	-2.32	0.07	BRE antisense RNA 1
DOK6	-2.25	0.10	docking protein 6
NME8	-2.21	0.06	NME/NM23 family member 8
AOAH-IT1	-2.21	0.09	AOAH intronic transcript 1 (non-protein coding)
STK38	-2.15	0.06	serine/threonine kinase 38
AKT3	-2.11	0.05	v-akt murine thymoma viral oncogene homolog 3
CARD8	-1.96	0.05	caspase recruitment domain family, member 8
PTPRJ	-1.94	0.08	protein tyrosine phosphatase, receptor type, J
RNU6-878P	-1.93	0.10	RNA, U6 small nuclear 878, pseudogene
AOAH	-1.93	0.06	acyloxyacyl hydrolase (neutrophil)
HIST1H2BI	-1.88	0.06	histone cluster 1, H2bi
RP11-269F20.1	-1.79	0.10	putative novel transcript
TRGV8	-1.77	0.06	T cell receptor gamma variable 8
FCRL6	-1.76	0.09	Fc receptor-like 6
VNN2	-1.74	0.06	vanin 2
WHSC1L1	-1.73	0.09	Wolf-Hirschhorn syndrome candidate 1-like 1
NLK	-1.66	0.10	nemo-like kinase
ZNF827	-1.54	0.08	zinc finger protein 827
ARHGAP15 // LOC101928361	-1.52	0.10	Rho GTPase activating protein 15 // uncharacterized LOC101928361
AF131217.1	-1.43	0.06	novel transcript
TSPAN3	-1.40	0.07	tetraspanin 3
IKZF2	-1.37	0.07	IKAROS family zinc finger 2 (Helios)
RNU6-628P	-1.36	0.06	RNA, U6 small nuclear 628, pseudogene
LOC202025	-1.35	0.07	uncharacterized LOC202025
FRYL	-1.30	0.07	FRY-like
RNA5SP390	-1.29	0.08	RNA, 5S ribosomal pseudogene 390
BBS9	-1.24	0.06	Bardet-Biedl syndrome 9
IGF1R	-1.18	0.06	insulin-like growth factor 1 receptor
ZNF43	-1.18	0.10	zinc finger protein 43
KIF2A	-1.17	0.10	kinesin heavy chain member 2A
LINC00264	-1.15	0.06	long intergenic non-protein coding RNA 264
ABCA5	-1.12	0.06	ATP-binding cassette, sub-family A (ABC1), member 5
CDK8	-1.10	0.10	cyclin-dependent kinase 8
DGKK	-1.09	0.09	diacylglycerol kinase, kappa
MRPS14	-1.08	0.10	mitochondrial ribosomal protein S14
ZMYM1	-1.05	0.10	zinc finger, MYM-type 1
MGAT5	-1.05	0.06	mannosyl (alpha-1,6-)-glycoprotein beta-1,6-N-acetyl-glucosaminyltransferase
RP11-101E14.2	-1.04	0.07	putative novel transcript
ZBTB20	-1.03	0.08	zinc finger and BTB domain containing 20
KLHDC10	-1.00	0.08	kelch domain containing 10
SNORD107	-0.95	0.06	small nucleolar RNA, C/D box 107
FLJ44635	-0.93	0.10	TPT1-like protein
CNOT6	-0.92	0.07	CCR4-NOT transcription complex, subunit 6
MBD5	-0.92	0.09	methyl-CpG binding domain protein 5
SNORD19	-0.90	0.06	small nucleolar RNA, C/D box 19
MYO9A	-0.89	0.10	myosin IXA
AC079584.2	-0.88	0.06	novel transcript
STIM1	-0.88	0.10	stromal interaction molecule 1
RP4-798A10.4	-0.87	0.09	novel trancript
KCNJ1	-0.87	0.06	potassium inwardly-rectifying channel, subfamily J, member 1
TBCK	-0.85	0.07	TBC1 domain containing kinase
SEPT1.	-0.83	0.06	septin 1
UBE2Q2P1	-0.82	0.08	ubiquitin-conjugating enzyme E2Q family member 2 pseudogene 1
IMMP1L	-0.79	0.10	IMP1 inner mitochondrial membrane peptidase-like (S. cerevisiae)
ACADSB	-0.78	0.10	acyl-CoA dehydrogenase, short/branched chain
MBNL3	-0.75	0.07	muscleblind-like splicing regulator 3
ACO1	-0.74	0.10	aconitase 1, soluble
FAM172A	-0.72	0.10	family with sequence similarity 172, member A
ZMYND8	-0.70	0.10	zinc finger, MYND-type containing 8
LOC389765	-0.66	0.07	kinesin family member 27 pseudogene
UST	-0.65	0.10	uronyl-2-sulfotransferase
BBIP1	-0.65	0.06	BBSome interacting protein 1
RP11-381K20.2	-0.64	0.10	novel transcript
FUT8	-0.63	0.06	fucosyltransferase 8 (alpha (1,6) fucosyltransferase)
LOC101927150	-0.61	0.09	uncharacterized LOC101927150
NEO1	-0.58	0.07	neogenin 1
ZMAT1	-0.53	0.07	zinc finger, matrin-type 1
FOCAD	-0.49	0.10	focadhesin

Supplementary Table 1 The genes with differential expression between HD-NK and D14-NK from the microarray analysis with an adjusted p-value of <=0.1.

			-Log(FDR)	
**Gene Set**	**Mean log_2FC**	**Gene set size**	**UP in NK-14**	**Down in NK-14**
KEGG_NATURAL_KILLER_CELL_MEDIATED_CYTOTOXICITY	-4.50	133	0.00	3.73
REACTOME_REGULATION_OF_IFNG_SIGNALING	-1.15	13	0.00	0.48
PID_IFNG_PATHWAY	-3.24	40	0.00	1.97
GO_NEGATIVE_REGULATION_OF_INTERFERON_GAMMA_PRODUCTION	1.12	35	0.15	0.00
GO_REGULATION_OF_INTERFERON_GAMMA_PRODUCTION	-0.95	93	0.00	0.26
GO_INTERFERON_GAMMA_PRODUCTION	-0.97	15	0.00	0.26
GO_POSITIVE_REGULATION_OF_INTERFERON_GAMMA_PRODUCTION	-1.52	61	0.00	0.47
GO_NEGATIVE_REGULATION_OF_TUMOR_NECROSIS_FACTOR_SUPERFAMILY_CYTOKINE_PRODUCTION	1.37	43	0.21	0.00
GO_POSITIVE_REGULATION_OF_TUMOR_NECROSIS_FACTOR_SUPERFAMILY_CYTOKINE_PRODUCTION	-1.18	56	0.00	0.34
GO_NATURAL_KILLER_CELL_ACTIVATION	-1.71	53	0.00	0.56
GO_POSITIVE_REGULATION_OF_NATURAL_KILLER_CELL_ACTIVATION	-1.20	18	0.00	0.35
NK_RELATED_TRANSCRIPTION_FACTORS	-1.51	10	0.03	1.12

Supplementary Table 2: Gene set enrichment analysis of NK cell physiology pathways. The gene set enrichment analysis was carried out using various gene sets, including cytotoxicity pathway, IFNG production pathway, TNF productionin pathway and NK related transcription factors. All of these gene sets are obtained from MSigDB(http://software.broadinstitute.org/gsea/msigdb/index.jsp) other than NK_RELATED_TRANSCRIPTION_FACTORS,(inculding: E4BP4, ID2, BLIMP1, EOMES, ETS-1, GATA-3, HELIOS, KLF4, T-bet and TOX) which is gathered from a review (*Frontiers in Immunology*. 2012;3:319. doi:10.3389/fimmu.2012.00319.). The FDR is the false discovery rate (the adjusted p value) and anything with FDR <=0.1 (or -Log(FDR)>=1) is considered a significant enrichment of the genes in the gene set.

**D7**	**Area**	**Standard error**	**Asymptotic significance**	**Asymptotic 95% CI**
**total NK**	**0.683**	**0.072**	**0.021**	**0.541-0.824**
**CD56bright**	**0.673**	**0.072**	**0.029**	**0.531-0.815**
**CD56dim**	**0.678**	**0.073**	**0.024**	**0.535-0.822**
**NKG2A**	**0.696**	**0.073**	**0.013**	**0.553-0.839**
**NKG2C**	**0.659**	**0.069**	**0.045**	**0.523-0.794**
**CD57**	**0.708**	**0.068**	**0.009**	**0.574-0.841**
**KIR**	**0.694**	**0.072**	**0.014**	**0.553-0.836**
**CD16**	**0.678**	**0.080**	**0.042**	**0.522-0.834**

**D14**	**Area**	**Standard error**	**Asymptotic significance**	**Asymptotic 95% CI**
**total NK**	**0.657**	**0.073**	**0.035**	**0.513-0.801**
CD56bright	0.635	0.069	0.07	0.5-0.771
**CD56dim**	**0.664**	**0.074**	**0.028**	**0.518-0.809**
**NKG2A**	**0.667**	**0.069**	**0.025**	**0.532-0.801**
**NKG2C**	**0.655**	**0.072**	**0.038**	**0.514-0.796**
CD57	0.610	0.075	0.139	0.464-0.757
KIR	0.638	0.078	0.069	0.486-0.790
CD16	0.647	0.088	0.085	0.474-0.820

**D28**	**Area**	**Standard error**	**Asymptotic significance**	**Asymptotic 95% CI**
total NK	0.507	0.163	0.965	0.188-0.825
CD56bright	0.573	0.163	0.631	0.253-0.894
CD56dim	0.480	0.146	0.896	0.194-0.766
NKG2A	0.547	0.161	0.760	0.232-0.862
NKG2C	0.440	0.157	0.694	0.132-0.748
CD57	0.480	0.151	0.896	0.184-0.776
KIR	0.480	0.165	0.896	0.158-0.802
CD16	0.593	0.197	0.644	0.207-0.978

**D100**	**Area**	**Standard error**	**Asymptotic significance**	**Asymptotic 95% CI**
total NK	0.434	0.153	0.685	0.135-0.734
CD56bright	0.316	0.142	0.256	0.037-0.595
CD56dim	0.408	0.159	0.570	0.096-0.720
NKG2A	0.316	0.127	0.256	0.067-0.565
NKG2C	0.474	0.181	0.871	0.119-0.828
CD57	0.434	0.162	0.685	0.116-0.752
KIR	0.421	0.160	0.626	0.108-0.734
CD16	0.434	0.166	0.685	0.108-0.760

Supplementary Table 3: An assessment of the ability of absolute NK cell number and NK subset number at varying time points to predict acute GVHD. Receiver operating characteristic curve analysis.
