# Supplementary material for: NK cells produce high levels of IL‐10 early after allogeneic stem cell transplantation and suppress development of acute GVHD
Source: Eur J Immunol. 2017 Oct 27;48(2):316–29. doi: 10.1002/eji.201747134 (PMC5836991; doi:10.1002/eji.201747134)
Supplement: Supplementary file 1 — Peer review correspondence [file EJI-48-316-s001.pdf]

European Journal of Immunology

NK cells produce high levels of IL-10 early after allogeneic stem cell transplantation and suppress development of acute GVHD

YL Tracey Chan, Jianmin Zuo, Charlotte Inman, Wayne Croft, Jusnara Begum, Joanne Croudace, Francesca Kinsella, Luke Maggs, Sandeep Nagra, Jane Nunnick, Ben Abbotts, Charles Craddock, Ram Malladi, Paul Moss

.Correspondence: Prof. Paul Moss, Institute of Immunology and Immunotherapy, United Kingdom of Great Britain and Northern Ireland

---

Review Timeline:	Submission date:	16-May-2017
	First Editorial decision:	5-Jun-2017
	Revision received:	16-Aug-2017
	Accepted:	18-Sep-2017

Handling Executive Committee member: Prof. James Di Santo

Please note that the correspondence below does not include the standard editorial instructions regarding preparation and submission of revised manuscripts, only the scientific revisions requested and addressed.

First Editorial Decision
15-Jun-2017

Dear Dr. Zuo,

Manuscript ID eji.201747134 entitled "NK cells produce high levels of IL-10 early after allogeneic stem cell transplantation and suppress development of acute GVHD" which you submitted to the European Journal of Immunology has been reviewed. The comments of the referees are included at the bottom of this letter.

A revised version of your manuscript that takes into account the comments of the referees will be reconsidered for publication. Should you disagree with any of the referees´concerns, you should address this in your point-by-point response and provide solid scientific reasons for why you will not make the requested changes.

You should also pay close attention to the editorial comments included below. **In particular, please edit your figure legends to follow Journal standards as outlined in the editorial comments. Failure to do this will result in delays in the re-review process.**

Please note that submitting a revision of your manuscript does not guarantee eventual acceptance, and that your revision will be re-reviewed by the referees before a decision is rendered.

If the revision of the paper is expected to take more than three months, please inform the editorial office. Revisions taking longer than six months may be assessed by new referee(s) to ensure the relevance and timeliness of the data.

Once again, thank you for submitting your manuscript to European Journal of Immunology and we look forward to receiving your revision.

Yours sincerely,
Nadja Bakocevic

On behalf of
Prof. James Di Santo

Dr. Nadja Bakocevic
Editorial Office
European Journal of Immunology
e-mail: ejied@wiley.com
www.eji-journal.eu

****

Reviewer: 1

Comments to the Author
Dr. Chan et al, here present NK cell specific immune reconstitution data from 82 patients after allo-HCT receiving in vivo T cell depletion with alemtuzumab (majority) or ATG.

I am very impressed by their NK cell reconstitution and functional data. Inverse relationship with aGVHD is also interesting.

European Journal of Immunology

I have following questions/suggestions for the authors:

1. How do they explain their the very impressive reconstitution of mature NK cells (CD56dim, KIR+) cells when everyone else has shown that the NK cells reconstituting early after allo-HCT are mostly hypo-functional (esp IFNg) CD56brights?

2. Why are these cells producing IFNg, IL-10 and TNF at baseline (without stimulation) on day 14, what is the trigger for this cytokine production in these cells?

3. I strongly suggest adding functional data (IL-10, IFNg, TNF with and without K562 stimulation) on day 28/30 samples. Is it possible that these cells on day 7/14 are graft derived cells which are replaced by more immature and hypo-functional NK cells derived from the graft CD34+ cells?

4. Suggest adding subset analysis looking at risk of relapse and NK cells data in their AML cohort.

5. Is their data unique to the patients receiving in vivo T cell depletion? Strongly suggest adding 10-12 day 7, 14 and day 30 samples for NK cell reconstitution and functional data from patients who receive non T cell depleted (no ATG or Alemtuzumab) transplants.

6. Suggest deleting the paragraph in their intro section which talks about post transplant cyclophosphamide as none of these patients received it.

Reviewer: 2

Comments to the Author
The manuscript by Chan T. et al. analyzed NK cell reconstitution at the early time after allogeneic hematopoietic stem cell transplantation (allo-HSCT). This work analyzed a cohort of 82 allo-HSCT patients. The authors described that NK cell reconstitution two weeks after HSCT is based on the homeostatic proliferation of donor NK cells with high capacities to produce large amounts of IL-10. Moreover, high numbers of NK cells 14 days post-HSCT are highly associated with a reduced risk of acute GvHD.

Major comments
- NKG2C and CMV status: the authors claimed that the balance between NKG2A and NKG2C expressions is related to the maturity of the NK cell populations. However, it is broadly described that NKG2C expression is mainly related to the CMV status. Did the authors stratify donors and recipients

according to CMV serology? What is the expression of NKG2C in recipients in D+/R- or D+/R+ pairs compared to D-/R+? Is there any relationship with the level of expansion observed in patients?
- The authors presented transcription profiles for NK-14 cells compared to NK cells from HD, however results seemed unexpectedly inconclusive. Results concerning pathways involved in NK cell physiology could be at least cited: cytotoxicity, IFNg production, TNF production, NK-related transcription factors (T-bet, Eomesâ€¦)... Given that NK-14 cells produce high amounts of IL-10, a special emphasis on this pathway would be welcome. Given the low fold change observed for the IL-10 transcript compared to the one observed for the protein, this result needs to be confirmed on more samples, at least by qRT-PCR.
- According to the authors, NK cells are the main lymphocyte population during the early period post-HSCT and the vast majority of those cells produce high amounts of IL-10. Therefore, what is the quantity of IL-10 in patients at 14 days post HSCT? Does it correlate with the NK cell number and with patient's outcome, or with other cytokines produced by or necessary to NK cellsâ€¦? What is the NK-related cytokine landscape in patients at day 14?

Minor comments:
- Bibliographic citations do not match with Eur J Immunol recommendations.
- Greek characters are not correctly depicted in Figure 1 (y-axis, panels A and B) and Figure 3 (x-axis, panels D, E and F).
- Size of Figures 2D and 2E should be increased as they are hardly readable.
- Figure 3D. There is no logic in the order of the combination of cytokines secreted by NK-14 cells. This legend should be reorganized.
- Figure 4B. It is impossible to read gene's names listed in the heatmap. The quality of the figure must be ameliorated.

First Revision – authors' response
16-Aug-2017

Reviewer #1
1. How do they explain their the very impressive reconstitution of mature NK cells (CD56dim, KIR+) cells when everyone else has shown that the NK cells reconstituting early after allo-HCT are mostly hypo-functional (esp IFNg) CD56brights?
Thank you for this comment. We agree that NK reconstitution is impressive although absolute NK cell numbers remain below normal values at day 14. Previous reports of NK reconstitution following allo-HCT have focused on later time points, typically day 28-30 after transplant, rather than day 14 value which was addressed in our study and is the peak time in relation to the proportion of CD56dim NK cells. In addition, most previous studies relate to 'T cell replete' transplants rather than the T cell depleted regiment that was used in our patient cohort and this may act to modulate the phenotype and function of NK cells in the early

post-transplant period.

2. Why are these cells producing IFNg, IL-10 and TNF at baseline (without stimulation) on day 14, what is the trigger for this cytokine production in these cells?

We agree that this is a surprising finding but we would also point out that we see the same observation for T cells at this timepoint. Lymphoid cells are undergoing intense homeostatic and antigen-driven proliferation in the early post transplant period and this, coupled with the inflammation resulting from the conditioning regimen, appears to drive cells to this novel phenotype. It is noteworthy that this pattern of 'spontaneous' cytokine production has also been observed in murine NK cells during homeostatic proliferation.

3. I strongly suggest adding functional data (IL-10, IFNg, TNF with and without K562 stimulation) on day 28/30 samples. Is it possible that these cells on day 7/14 are graft derived cells which are replaced by more immature and hypo-functional NK cells derived from the graft CD34+ cells?

Thank you for this. We have now performed these experiments and the result shows that NK cells at day 28 are indeed less functional than NK cells from healthy donors after K562 cell stimulation. Interesting, however, NK cells from day 28 after HSCT continue to

spontaneously produce more IL-10 than healthy donors (0.34% vs 0.06% $P<0.05$) although the percentage of IL-10-producing cells is markedly reduced compared with NK cells at day14 (supplementary figure 4).

4. Suggest adding subset analysis looking at risk of relapse and NK cells data in their AML cohort.

This was assessed by plotting receiver operating curves. A p-value of <0.05 was used as the threshold for statistical significance.

D7: p= 0.059; AUC 0.676 (n=43)

D14: p= 0.553; AUC 0.553 (n=43)

D28: p= 0.019; AUC 0.958 (n=10)

D100: p= 0.048; AUC 0.955 (n=13)

As such we did not find that D14-NK cell number was predictive of relapse in the AML/MDS patient cohort. This data does suggest NK cell number at day D28 and D100 is predictive of relapse although the number of patients in these subgroups is modest.

5. Is their data unique to the patients receiving in vivo T cell depletion? Strongly suggest adding 10-12 day 7, 14 and day 30 samples for NK cell reconstitution and functional data from patients who receive non T cell depleted (no ATG or Alemtuzumab) transplants.

This is an interesting question and we have performed this analysis. We find that the absolute NK cell number at D7 and D14 is comparable in both T-replete (TR) and T cell-depleted (TCD) transplants. However, there is an interesting contrast in the pattern of NK cell subset reconstitution between these two time points. The absolute number of CD56bright NK cells at D14 is higher in TR transplants compared to TCD transplants (p>0.001). Furthermore, when expressed as a proportion of the total NK cell population, the CD56bright NK cell subset in TR transplants in significantly larger than that seen in TCD transplants at

D14 (p<0.001). The inverse is seen for the CD56dim NK cell subset.(Supplementary Figure 2). As the Reviewer suggests, the nature of the conditioning regimen can make an important influence on immune reconstitution and these points have been added to the discussion.

6. Suggest deleting the paragraph in their intro section which talks about post transplant cyclophosphamide as none of these patients received it.

Done

Reviewer: #2

Major Comment:

1. NKG2C and CMV status: Did the authors stratify donors and recipients according to CMV serology? What is the expression of NKG2C in recipients in D+/R- or D+/R+ pairs compared to D-/R+? Is there any relationship with the level of expansion observed in patients?

A B

Rebuttal Figure 1: The percentage of NKG2C (A) positive NK cells and total count of NK cells (cells/ul) (B) were compared between different groups according to their CMV status of patients and donors (D+R- (n=15) means CMV positive donors with CMV negative patients; D+R+ (n=33) means CMV positive donors with CMV positive patients; D-R+ (n=25) means CMV negative donors with CMV positive patients; D-R- (n=9) means CMV negative donors with CMV negative patients) . The data is shown as mean +/- SEM and the statistical significance between the four groups was assessed using a one-way ANOVA.

This is an interesting question as CMV has an important influence on the NK repertoire. The data in rebuttal figure 1A show that CMV serostatus has no statistically significant influence on the percentage of NKG2C-positive NK cells (as a proportion of the total NK cell population) at D14 following allo-SCT between the four groups. This may reflect the relatively small numbers in each group.

The data in rebuttal figure 1B show that the mean number of NK cells appears higher when both donor and recipient are CMV seropositive but this difference is not statistically significant due to significant variation between patients and the relatively small number in each subgroup.

2. The authors presented transcription profiles for NK-14 cells compared to NK cells from HD, however results seemed unexpectedly inconclusive. Results concerning pathways involved in NK cell physiology could be at least cited: cytotoxicity, IFNg production, TNF production, NK-related transcription factors (T-bet, Eomes…)...

Thank you. Microarray analysis which addresses several NK cell pathways, including cytotoxicity, IFN□ production, TNF production and NK-related transcription factors, has now been added. (Supplementary table 2). All of these gene sets are obtained from MSigDB apart from NK transcription factors which developed from Luevano, et al. Frontiers in Immunology. 2012;3:319). A significant downregulation of NK

cytotoxicity pathways, the IFN□pathway and NK-related transcription factors is observed.

3. Given the low fold change observed for the IL-10 transcript compared to the one observed for the protein, this result needs to be confirmed on more samples, at least by qRT-PCR.

We agree this is an important point. The qRT-PCR has now been performed with NK cells at day 14 after HSCT. Interestingly, the transcription of IL-10 is around 6 times higher compared to healthy donors. (new figure 4E)

4. What is the quantity of IL-10 in patients at 14 days post HSCT? Does it correlate with the NK cell number and with patient's outcome, or with other cytokines produced by or necessary to NK cells…? What is the NK-related cytokine landscape in patients at day 14?

The concentration of IL-10, TNF-a and IFN-g in the serum of day14 patients after HSCT has now been studied and compared with healthy donors. The result shows that both the IL-10 and TNF-a levels are around 4-fold higher in the serum of day 14 patients compared to healthy donors. In contrast, the concentration of IFN□was not increased. We did attempt to correlate IL-10, TNF-□and IFN-□ concentration with the incidence of aGvHD but no such association was observed (Supplementary figure 3).

Minor comments:

1. Bibliographic citations do not match with Eur J Immunol recommendations.

This has now been corrected

2. Greek characters are not correctly depicted in Figure 1 (y-axis, panels A and B) and Figure 3 (x-axis, panels D, E and F).

Corrected

3. Size of Figures 2D and 2E should be increased as they are hardly readable.

Corrected

4. Figure 3D. There is no logic in the order of the combination of cytokines secreted by NK-14 cells. This legend should be reorganized.

Thank you. The order of the cytokines was arranged according the percentage of NK cell production, with the highest values first. In Figure 3E the logic of the order is firstly IL-10 neg/pos, then IFN-γ neg/pos and at last TNF-α pos/neg.

5. Figure 4B. It is impossible to read gene's names listed in the heatmap. The quality of the figure must be ameliorated.

Corrected

<u>Second Editorial Decision</u>

<u>04-Sep-2017</u>

Dear Dr. Zuo,

It is a pleasure to provisionally accept your manuscript entitled "NK cells produce high levels of IL-10 early after allogeneic stem cell transplantation and suppress development of acute GVHD" for publication in the European Journal of Immunology. For final acceptance, please follow the instructions below and return the requested items as soon as possible as we cannot process your manuscript further until all items listed below are dealt with.

Please note that EJI articles are now published online a few days after final acceptance (see Accepted Articles: http://onlinelibrary.wiley.com/journal/10.1002/(ISSN)1521-4141/accepted). The files used for the Accepted Articles are the final files and information supplied by you in Manuscript Central. You should therefore check that all the information (including author names) is correct as changes will NOT be permitted until the proofs stage.

We look forward to hearing from you and thank you for submitting your manuscript to the European Journal of Immunology.

Yours sincerely,
Marta Vuerich

on behalf of
Prof. James Di Santo

Dr. Marta Vuerich
Editorial Office
European Journal of Immunology
e-mail: ejied@wiley.com
www.eji-journal.eu
